# NeuroActivityToolkit—Toolbox for Quantitative Analysis of Miniature Fluorescent Microscopy Data

**DOI:** 10.3390/jimaging9110243

**Published:** 2023-11-06

**Authors:** Evgenii Gerasimov, Alexander Mitenev, Ekaterina Pchitskaya, Viacheslav Chukanov, Ilya Bezprozvanny

**Affiliations:** 1 Laboratory of Molecular Neurodegeneration, Peter the Great St. Petersburg Polytechnic University, Khlopina St. 11, 194021 St. Petersburg, Russia; 2 Department of Physiology, UT Southwestern Medical Center at Dallas, Dallas, TX 75390, USA

**Keywords:** miniscope, Minian, software, miniature fluorescence microscopy, statistical analysis, metrics, open-source toolbox

## Abstract

The visualization of neuronal activity in vivo is an urgent task in modern neuroscience. It allows neurobiologists to obtain a large amount of information about neuronal network architecture and connections between neurons. The miniscope technique might help to determine changes that occurred in the network due to external stimuli and various conditions: processes of learning, stress, epileptic seizures and neurodegenerative diseases. Furthermore, using the miniscope method, functional changes in the early stages of such disorders could be detected. The miniscope has become a modern approach for recording hundreds to thousands of neurons simultaneously in a certain brain area of a freely behaving animal. Nevertheless, the analysis and interpretation of the large recorded data is still a nontrivial task. There are a few well-working algorithms for miniscope data preprocessing and calcium trace extraction. However, software for further high-level quantitative analysis of neuronal calcium signals is not publicly available. NeuroActivityToolkit is a toolbox that provides diverse statistical metrics calculation, reflecting the neuronal network properties such as the number of neuronal activations per minute, amount of simultaneously co-active neurons, etc. In addition, the module for analyzing neuronal pairwise correlations is implemented. Moreover, one can visualize and characterize neuronal network states and detect changes in 2D coordinates using PCA analysis. This toolbox, which is deposited in a public software repository, is accompanied by a detailed tutorial and is highly valuable for the statistical interpretation of miniscope data in a wide range of experimental tasks.

## 1. Introduction

Visualization of the neuronal activity within a specific brain area in a freely moving animal in vivo allows researchers to obtain an extensive array of information about neuronal activation patterns, changes in their excitability and their connections between each other [1,2]. Canonical methods for examining brain neuronal activity are two-photon microscopy [3,4,5,6] and electrode arrays [7,8,9,10]. Recently, miniature fluorescent microscopy (miniscope) was invented [11,12], which enables the fluorescent imaging of neurons’ activations in vivo in freely moving mice. Since its implementation in the neurosciences, miniscope imaging has become a powerful and independent tool for investigating the functions of neuronal circuits and complex neuronal networks [13,14]. Moreover, the miniscope gives neurobiologists a wide range of capabilities to explore neuropathology or neurodegenerative disorders in the context of the neuronal network state.

To visualize neuronal activity utilizing a miniscope, different genetically encoded calcium indicators are employed, of which the GCaMP family is the most popular [3,15]. The primary miniscope data consist of 752 × 480 resolution videos for the v3 version, containing information about the location of cells and calcium-indicator changes over time. Yet, recordings have fluctuating background and motion artifacts resulting from the movement of mice and the technical properties of the miniscope, making the consequent extraction of individual neuron calcium activity challenging [16,17,18]. For processing this quite-noisy data, several open-source protocols have been developed [19,20,21,22]. In short, these methods aim to reduce the influence of the background on single-cell calcium activity, extract and denoise calcium intensity changes over time and merge overlapping units during the recording session [23]. In the current study, the Minian software method was used for preprocessing [19], since it has an easy-to-start pipeline and simple workflow integrated in the development environment “Jupyter Notebook”.

In this paper, the NeuroActivityToolkit software package is presented for the further quantitative analysis of the calcium activity extracted with Minian individual neurons. This toolbox offers a wide range of quantification of statistical metrics to describe neuronal network properties. It has a clear interface, visible workflow in the “Jupyter Notebook” environment, and obtained results can be extracted in .xlsx format for further statistical processing. Analysis of miniscope data using NeuroActivityToolkit starts by uploading miniscope data preprocessed with Minian [19] or other compatible file formats with proper hierarchy. Once the calcium traces are uploaded, the user chooses the part of the signal to be analyzed—a phase of rapid fluorescent-intensity growth or a phase above the adaptive threshold. Then, NeuroActivityToolkit calculates statistical metrics describing the activity of individual neurons as well as the entire neuronal network. It also offers an option for analyzing neuronal activity correlations and measuring the distance between co-active cells. All metrics are subjected to PCA analysis to characterize the neuronal network state in 2D coordinates and visualize it. Moreover, a detailed tutorial is presented visualizing the most critical steps to ensure an easy work start with the toolbox. Therefore, this paper offers a comprehensive open-source software package for the analysis of neuronal activity extracted from miniscope data.

## 2. Materials and Methods

### 2.1. Mice

An FVB breeding colony of mice obtained from the Jackson Laboratory (Bar Harbor, ME, USA) was established and maintained in a vivarium with 4–5 mice per cage and a 12 h light/dark cycle in the animal facility, with ad libitum access to food and water. All procedures were approved by principles of the European convention (Strasburg, France, 1986) and the Declaration of International Medical Association regarding the humane treatment of animals (Helsinki, Finland, 1996), and approved by the Bioethics Committee of the Peter the Great St. Petersburg Polytechnic University at St. Petersburg, Russia (Ethical permit number 1-n-b from 3 February 2023). In the current research, data from a 9-month-old mouse were used to validate the toolbox, and data from other mice of the same age to illustrate the PCA method.

### 2.2. Implantation of GRIN-Lens and Baseplate for Miniscope Recordings

Implantation of the GRIN-lens was performed in two-stages. Firstly, viral construct pAAV1.Syn.GCaMP6S.WPRE.SV40 at a titer of more than 1 × 10^13^ vg/mL was injected with AP −2.1; DV −1.8; ML +2.1 stereotaxic coordinates (68001, RWD Life Science, Shenzhen, China) into the left hemisphere under isoflurane anesthesia, using the standard protocol [24]. Viral construct of a total volume of 1.4 µL was injected at a rate of 0.1 µL per minute. After 3 weeks, a gradient index lens 2 mm in diameter was implanted via the protocol described in [25]. Four weeks after stereotaxic injections, the baseplate was fixed on the mice’s heads in a position to obtain the best ROI of hippocampal neurons expressing GCaMP6s. Then, after recovery from surgery and isoflurane exposure, 5 min in vivo imaging was performed using a miniscope v3 (Labmaker, Berlin, Germany) once a weak in the home-cage condition in freely moving mice. In the days of the recordings, the miniscope was fixed to the baseplate without any anesthesia exposure. The number of recorded neurons equaled 142 ± 29.

### 2.3. Miniscope Recording Acquisition and Preprocessing

Miniscope data were recorded via the free-to-access Portable Miniscope Data Acquisition program (Pomidaq, version number 0.4.5) at 20 frames per second. All the recordings were obtained in the “mkv” format. Further processing of the miniscope data was performed using Minian [19] with standard parameters for CNMF method inside the pipeline and the following characteristics for the initialization parameters: “wnd_size”: 1000, “method”: “rolling”, “stp_size”: 500, “max_wnd”: 15, “diff_thres”: 3. This method formed an array containing Ca^2+^ indicator fluorescence traces and the location of each detected neuron. Subsequent quantitative analysis of the miniscope data was performed using the NeuroActivityToolkit presented in this paper.

### 2.4. Neuron-Active-State Determination

As the first step for the quantitative analysis of neuronal network activity, the active states of the neurons were determined. The active state is a phase of the rapid growth of GCaMP6f fluorescence intensity, since action-potential generation leads to a robust elevation in intracellular calcium concentrations. Firstly, the preprocessed signals by Minian were smoothed by a moving average with a configurable window width:

(1) $$X_{t}=\frac{1}{n}\sum_{i=-[\frac{n}{2}]}^{i=[\frac{n}{2}]}p_{t-i},$$

where x_t_ is the resulting smoothed value at the point, n is the window width, and p_t−I_ is the initial value at the point t−i. Further, to determine the signal changes, a discrete derivative was calculated: x_t_′ = x_t_ − x_t−1_. This value shows how much the signal intensity changed at a particular time point. The segmentation of the already-preprocessed fluorescence signal to an active phase and inactive phase was based on a threshold value for the derivative. If this value exceeded the threshold, a neuron was assumed to be in the active state:

(2) $$threshold=median(x^{\prime })+mad(x^{\prime }),$$

where mad is the average absolute deviation. The median corresponds to the standard state of the neuron, while the average absolute deviation relates to the permissible variances. Thus, the active state of the neuron is assumed to be the time period when the signal intensity is higher than the permissible level. Detected active states could be analyzed in two different approaches: included in the analysis of both the signal stages (robust fluorescence growth and its decline) (*full method*) or only the rising part of the calcium-indicator fluorescence intensity (*spike active method*).

Additional segmentation settings were added to enhance the signal segmentation quality. *Warm*—the minimum duration of the passive phase: if the difference between neighboring active states is less than this value, they are combined into one active state. *Cold*—the minimum duration of the active phase; if it lasts for less time than the specified parameter, but exceeds the threshold value, it is excluded from the active state. These parameters are counted in frames.

### 2.5. Neuronal Network Description

The group of metrics describing neuronal activity was calculated based on the active states obtained in Section 2.4 [26]. Burst rate—the number of “cell activations” for a given time period. The average number of activations per unit of time is calculated as follows:

(3) $$burst\;rate\;=\frac{\sum_{x\in A}active(x)}{T},$$

where A is a set of neurons, active (x) equals 1 if this neuron is active; otherwise it is 0, and T is the time interval. The network spike rate is the percentage of active neurons over a specified time interval. The percent of active neurons in time interval is calculated as follows:

(4) $$network\;spike\;rate=\frac{\sum_{x\in A}active(x)}{size(A)},$$

where A is a set of neurons, active (x) equals 1 if this neuron is active; otherwise it is 0, and size equals the size of the set. The network spike duration is the length of time when the number of simultaneously active cells is higher than the preset threshold value:

(5) $$network\;spike\;duration=\frac{\sum_{i=1}^{T}\frac{\sum_{x\in A}active(x_{i})}{size(A)}>threshold}{T},$$

where active(x_i_) equals 1 if this neuron is active, and is otherwise 0, at the i-th moment of time, threshold is the established threshold value, and T is the time interval. The network spike peak is the maximal number of simultaneously active cells at a specified time interval:

(6) $$network\;spike\;peak={max}_{i=1}^{T}(\frac{\sum_{x\in A}active(x_{i})}{size(A)}).$$

### 2.6. Correlation Analysis for Co-Active Neurons

To analyze correlations between neuronal-activity values, Pearson’s coefficient was applied:

(7) $$r_{XY}=\frac{\sum_{i=1}^{N}\left(x_{i}-M_{X})(y_{i}-M_{Y}\right)}{\sqrt{{\sum_{i=1}^{N}(x_{i}-M_{X})}^{2}{\left(y_{i}-M_{Y}\right)}^{2}}}.$$

In the current toolbox, several ways for computing Pearson’s coefficient were presented. For the *original signal intensity*: X, Y—time scans of signal intensity, x_i_—the value of X at the i-th moment of time, y_i_—the value of Y at the i-th moment of time, M_X_—mean value of X, M_Y_—mean value for Y. For the *intensity derivative*: X, Y—time scans of signal intensity derivative. For the *binary results of active phase segmentation*: X, Y—time scans of active phase segmentation in binary form, where 1 = active, 0 = inactive (for *active spike* and *full* methods). Also, the *connection of intersection* is computed as the relation between the time when both neurons in the pair are simultaneously active to the sum of individual activities.

Next, we present the statistical metrics based on the obtained correlations. The network degree is the curve that shows the dependence of the number of correlating neuronal pairs on the preset threshold level. The abscissa axis indicates the threshold value of the correlation level; on the ordinate axis, the percentage of strong connections between all neurons satisfying the corresponding threshold value is indicated. An option for the “Lag” value was added for all metrics, which is the time shift between active phases of neurons when they are still considered as co-active.

The transfer of entropy from neuron X to another neuron Y is the amount of uncertainty reduced in future values of Y by taking into account the past values of X, providing the corresponding past values of Y:

(8) $$T_{X-Y}=I\left(Y_{t};X_{t-1:t-L}|Y_{t-1:t-L}\right)=H\left(Y_{t}|Y_{t-1:t-L}\right)+H\left(X_{t-1:t-L}|Y_{t-1:t-L}\right)-H(Y_{t},X_{t-1:t-L}|Y_{t-1:t-L}),$$

where X is the value of intensity for the “source” neuron, Y is the value of intensity for the “receiver” neuron, t is the moment of observation, L is the duration of analysis and H is the entropy.

Further, a special module was created to estimate the dependence between Pearson’s correlation coefficient and the distance between co-active neuronal pairs. The analysis was based on the dependence of Pearson’s correlation coefficient on the neuronal remoteness from the mass center of all neurons in the recording. The Euclidean and radial distance were used to estimate the distance between neurons. Also, it was used to count Pearson’s coefficient while considering the distance between neurons with the distance factor:

(9) $$k=\frac{dist}{dist+100},$$

where dist is the distance between co-active neurons in pixels, and 100 is the 25th percentile of the distance distribution values.

### 2.7. Neuronal-Activity Random Shuffling

This module is designed to assess the behavior of the provided metrics when different levels of randomness are introduced to the analyzed data. The differences between metrics counted for the original data and shuffled data may that indicate that they are capturing the neuronal network properties rather than independent neuronal-activity distribution. The mixing process was carried out as follows: the number of each neuron activations remained intact while the duration of active states and the time interval between them was randomly stated. The percent of shuffled neurons was determined via the shuffle ratio, that could be varied by the user needs. Thus, it was possible to track changes in statistical metric behavior at different levels of shuffling. Several independent iterations of shuffling events were performed to increase the algorithm robustness in the experiments.

### 2.8. PCA Analysis

PCA (principal component analysis) is a way to reduce the data dimensionality with minimal information loss. It is used to combine data into a two-dimensional space that is easy and convenient to visualize and analyze. For PCA analysis, all the statistical metrics mentioned above were used. There are two main approaches to calculating the principal components: using the eigenvalues and eigenvectors of covariance matrices, or singular value decomposition of the original matrix (SVD). In the current paper, a ready-made solution was used via an open-source Python library “scikit-learn” [27], which utilized the SVD method.

## 3. Results

### 3.1. Calcium-Indicator-Signal Active-Phase Determination

Miniscope data processed by Minian [19] included calcium-indicator traces and the location of each detected neuron. At the first step, it is important to determine time moments when the neuron is in an active state, characterized by a robust increase in the calcium indicator fluorescence intensity. The signal active-phase determination was performed in two stages. At first, the signal was smoothed via a moving average with a changeable window width (Supplementary Materials Section S3.1). The *wnd_size* parameter reflected the level of smoothing, ranging from 0 frames (no smoothing) to 50 frames (roughest way of smoothing). The appropriate value of *wnd_size* depended on the quality of the obtained calcium traces (the better it is, the smaller the parameter should be). Next, a discrete derivative was calculated to identify active states by evaluating a calcium indicator intensity shift at each time point. The derivative was measured between two consecutive frames and if its value was higher than the threshold level, threshold = median(x′) + mad(x′), the neuron was considered to be active at that time point. The median represented the average level of single-neuron activity, while the average absolute deviation contributed to permissible deviations. Further, all the neighboring frames were summed into the active state of the neuron.

For more precise signal segmentation into the active and non-active phases, two additional parameters were used: *warm*, which represents the minimum duration of the non-active phase, and *cold*, which represents the minimal duration of the active phase, both measured in frames. These parameters help to reject false-positive active states with single or short-lasting periods of activation, which are artifacts often detected in the recording (Figure S1). These settings can be adjusted to the experimental conditions, such as the recording parameters, types of calcium indicators or miniscope version. The influence of these parameters on the segmentation of the active phase is presented in Figure S1A,B. The window width parameter is significant for determining the active state in the relatively small calcium events (Figure S1C,D). By our observation, the most suitable values for *warm* were 15–50 frames, *cold* was 0 frames and *wnd_size* was 10. For the active-state detection of the sparsely active neurons, *wnd_size* can be set at 10 frames, but if there are lots of neurons with long-lasting periods of activity, it should be decreased as much as possible (from 1 to 5 frames). It can be manually adjusted by the user using an interactive widget.

**Figure 1:**
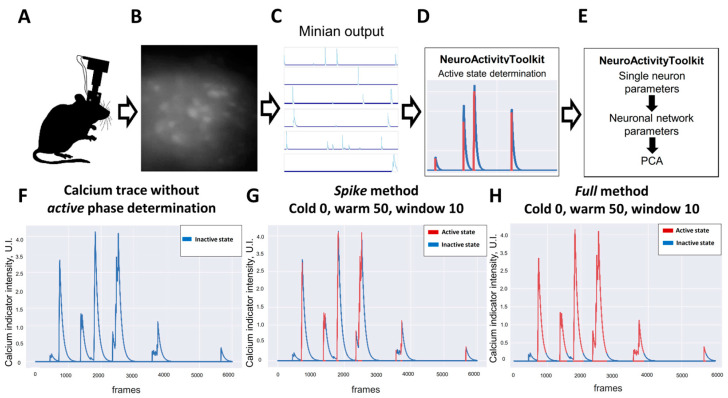
Pipeline of data processing using NeuroActivityToolkit. (**A**) Schematic illustration of the mouse with mounted version 3 miniscope. (**B**) Fluorescence of neurons in CA1 hippocampal area expressing GCaMP6s recorded via miniscope. (**C**) Calcium traces obtained from calcium recording processed with the Minian (version 1.2.1). (**D**) Active state determination as a first step of NeuroActivityToolkit pipeline. (**E**) Quantification of the miniscope recorded data in NeuroActivityToolkit toolbox. (**F**) Fluorescence intensity trace of the calcium indicator for the single neuron in the recording. Single-neuron active state determined using spike method (**G**) and full method (**H**). Active state is shown in red, inactive in blue.

All the metrics mentioned below are mostly based on active-phase extraction, so it is one of the most important steps for data processing using NeuroActivityToolkit. In addition to the *spike* method described above, where only the rising part of the calcium-indicator fluorescence intensity was included in the active state of the neuron (Figure 1G), we also present the *full* method, where all the signal above the threshold value was considered active (Figure 1H). In this manuscript, the spike method was used to determine an active state if the other was not mentioned.

### 3.2. Neuronal network Activity Properties

**Figure 2:**
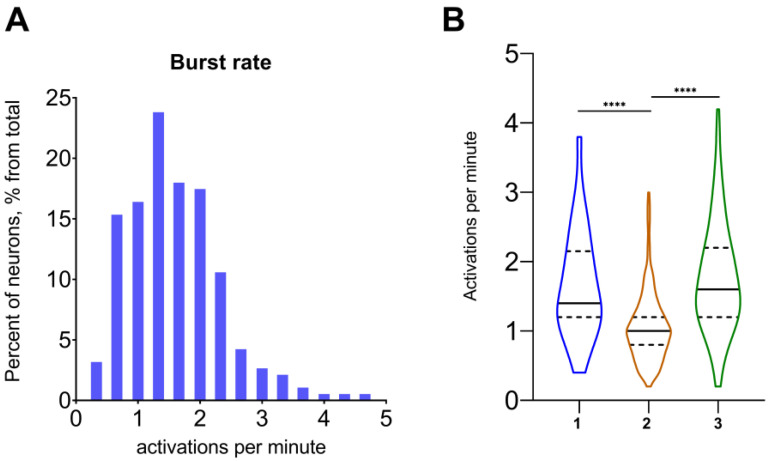
Activation properties for the example recording. (**A**) Distribution of number of activations per minute for neurons from an example recording. (**B**) Number of activations per minute for the independent recording from the same mouse, acquired on the 3 different days (1–3). Data on the B graph are presented as a violin plot with median (continuous line) and quartiles (dotted line). ****: *p* < 0.0001 (Kruskal–Wallis test with multiple comparisons using Dunn’s test).

The main purpose of the presented NeuroActivityToolkit is to provide information about neuronal activity at the single-neuron and neuronal network levels through the quantitative Miniscope data analysis. The statistical metric *Burst rate* was used to analyze the number of calcium events for the time interval for each neuron in the whole network (see Table 1 and Table 2). The distribution of the neuronal activation values for individual recording sessions is shown in Figure 2A. All single-neuron activations are saved as a “.xlsx” file. This representation format might be useful for identifying individual cell activation patterns (Figure 2B).

**Figure 3:**
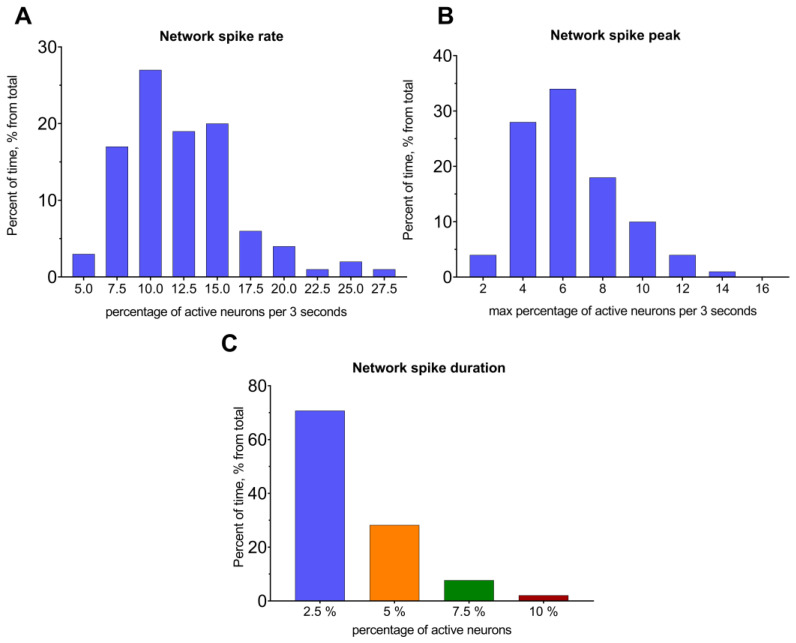
Neuronal network properties for the example recording. Distribution of network spike rate (**A**) and network spike peak (**B**) in the selected time interval of 3 s. Distribution for a single recording. (**C**) Distribution of network spike duration as a time when the amount of simultaneously active neurons was above the preset threshold value.

*The Network spike rate* (NSR) measures the percentage of active neurons within a specific time interval (Figure 3A and Table 1). The time interval for this metric can be varied from 1 s to 1 min. The *Network spike peak* (NSP) represents the maximum number of simultaneously active cells in the whole recording period for a certain interval of time (Figure 3B and Supplementary Materials Section S3.3). The *Network spike duration* (NSD) is a length of time when the number of active cells exceeds the predetermined threshold value (Figure 3C and Supplementary Materials Section S3.4).

These statistical metrics provide complex information about neuronal network status and are linked to the activation properties of neurons. Comparing them during experiments could help to evaluate overall changes in vivo in the different experimental conditions (Table 2). For example, if each metric exhibits a shift towards a higher value (a right shift), it may be associated with elevated excitability in the neuronal circuits.

### 3.3. Pairwise Neuronal-Activity Correlation in the Neuronal Network

**Figure 4:**
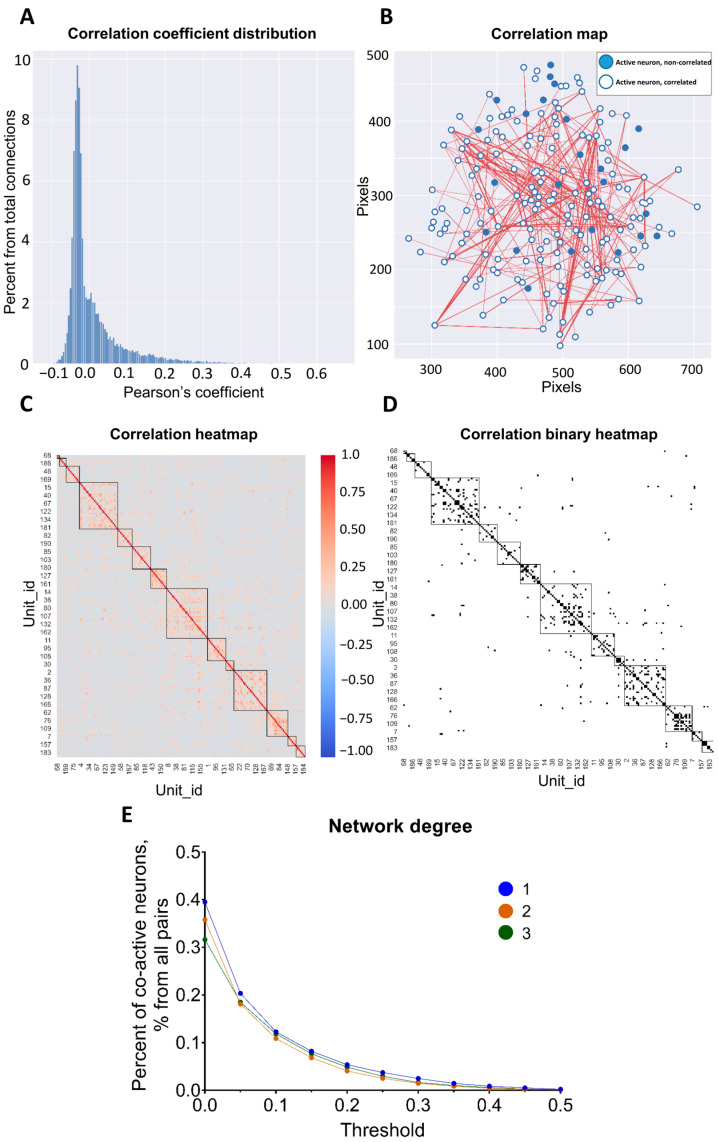
Neuronal-activity correlation analysis using Pearson’s coefficient. (**A**) Distribution of Pearson’s correlation coefficient. (**B**) Correlation map of co-active neuronal pairs. Correlated neurons are linked with the line between them in the space of the neuronal network. Only correlations above a 0.3 threshold value are shown. Axis values are indicated in pixels. (**C**) Correlation heatmap for connected pairs of neurons, from the highly negatively correlated in blue color to highly positively correlated in red color. Neurons are labeled by unit_id number. (**D**) Correlation heatmap in binary representation, where correlation above 0.3 threshold value is shown in black, and lower in white. For (**C**,**D**), clusters of closely related pairs of neurons are highlighted by squares. (**E**) Dependence of Pearson’s coefficient of correlation on the threshold level for 3 recordings for the same mouse (signal method).

Correlations between neuronal activity are a highly important measure of the neuronal activation similarity inside a network. Pearson’s correlation coefficient was used to detect the connections between pairs of neurons, indicating whether they had close activity patterns or were not “connected” at all. In the NeuroActivityToolkit, Pearson’s coefficient was computed in several ways depending on the input active-phase extraction, including the intensity of the original signal (*signal)*, the intensity of signal derivative (*diff)*, the segmented binary results (see Section 3.1) (*active* and *full* method), and the duration of simultaneously active states (*active_acc)* (Table 1, Figure S2 and Supplementary Materials Section S3.5). These correlation coefficients contained the most important co-activity characteristics of connected neuronal pairs. The correlation coefficient distribution for the *active* method is presented in Figure 4A. The correlation map for the determined method illustrates which neurons are connected in the network and their positions (Figure 4B). It shows the strength of connections between neurons, with positive connections represented in red and negative connections in blue (Figure 4C). Connections, in Figure 4C, are shown for neuronal pairs with correlation coefficient values above a threshold equaling 0.3, which can be easily set by a slider (Supplementary Materials Section S3.5). Values of the axes are given in the pixels. By setting a threshold value, the correlation binary heatmap highlighted connections that exceeded the defined threshold (Figure 4D). The choice of correlation coefficient calculation method was crucial for the accurate interpretation of the miniscope data (Figure S2 and Table 2). The less strict way is to use the *signal* method (Figure S2A,B), in which its correlation map is enriched with various strongly connected pairs of neurons, while the strictest method is *active_acc* (Figure S2G,H), in which only periods of simultaneous activity are examined [28]. Neurons were also clustered based on their correlation values, so that a pair of neurons belong to the same group if their connections are stronger than 80% of others in the network.

The correlation coefficient shares extended information about the intranetwork “behavior” of the neuronal pairs. Nevertheless, derivative statistics could also be useful for accurately interpreting the network state and providing helpful data for analysis (see Table 2). The *network degree* metric indicates the percentage of co-active neurons above the threshold level (Figure 4E). The threshold level was preset from 0 to 1 values with a 0.05 step. The data were saved in an “.xlsx” file (Table 1 and Supplementary Materials Section S3.6) for further analysis. According to our observations, the network degree was the most stable characteristic for individual mice within days of recordings.

**Figure 5:**
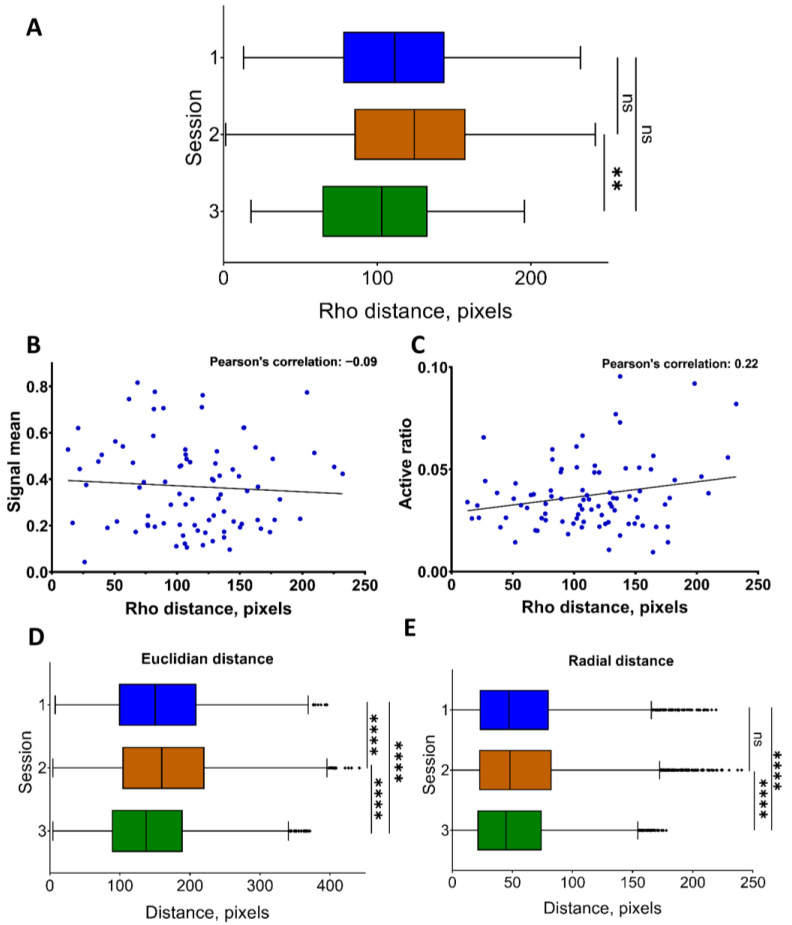
Spatial position of neuronal co-active pairs and Pearson’s correlation coefficient. (**A**) The distance to neurons from the center of their mass in polar coordinates (Rho) for 3 independent recordings. (**B**) Dependence between the detected signal mean fluorescence and distance in polar coordinates for each neuron in the recordings for a single miniscope recording. (**C**) Dependence between the active-state ratio (active state of neuron duration/total recording duration) and distance in polar coordinates for each recorded neuron for a single miniscope recording. Distance between all correlated neuronal pairs, as Euclidean (**D**) and radial (**E**) distance correspondingly, for 3 independent recordings. All the data are presented as the median values, borders of the box plots are 1 and 3 quartiles, and all the errors are interquartile ranges. ns—there were no significant differences, **: *p* < 0.01, ****: *p* < 0.0001 (Kruskal–Wallis test with multiple comparisons using Dunn’s test).

The “Distance analysis” module allows the user to estimate the dependence between the correlation coefficient and distance between neuronal pairs. The distance between pairwise connected neurons was calculated in the polar coordinates (the center of mass was computed between all neurons for each recording). The median value of the distances between co-active neuronal pairs in polar coordinates was presented as 1–3 quartiles in a box plot (see Figure 5A; the errors are the interquartile range (Supplementary Materials Section S4)). Scatterplots, presented in Figure 5B,C, were used to evaluate whether moving away from the center of the entire neuron mass affected the detection of the fluorescence signal when using the miniscope (Figure 5B,C). Pearson’s correlation coefficient shows that the level of connections between the stated characteristics and for the current dataset was relatively low: −0.19 ± 0.09 mean dependence of fluorescence intensity on the distance; 0.17 ± 0.03 for the dependence of the active state ratio on the distance in polar coordinates for both. These values can be found in the top right corner of the scatterplot for each recording.

Next, the median distance value was calculated for all neuronal pairs, regardless of the threshold level of correlation (Figure 5D,E). It could be set through a slider at the top of the section (Supplementary Materials Section S4). The distance of interest—*Euclidean* (the distance between pairs of co-active neurons—interneuronal distance) or *radial* (the difference in distances between pairs of neurons to the mass center of all neurons in the certain recording)—to the calculated above-described metrics was selected using the widget. Additionally, the user could choose the method by which correlations were calculated (signal, active, active_acc (intersection)) (Table 1 and Supplementary Materials Section S4). To facilitate subsequent analysis and plotting, all these statistics could be exported to tables in the “.xlsx” file format.

**Figure 6:**
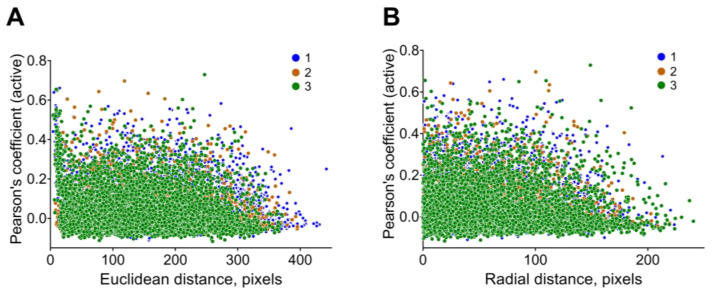
Distance between correlated neuronal pairs. Dependence between Euclidean distance (**A**) or radial distance (**B**) and Pearson’s coefficient for co-active neuronal pairs calculated using *active* (*spike*) method for 3 independent recordings.

The current module also provides a visual representation of the dependence of Pearson’s coefficient on the distance between the neuronal pairs. With the help of an interactive widget, users could select the values of interest. For example, Figure 6 shows the dependence of the correlation coefficient calculated based on the active states, using the active (spike) method from Euclidean coordinates (Figure 6A) and from the radial distance (Figure 6B).

Correlations between pairs of neurons inside the neuronal network are an essential part of normal brain functioning. Analyzing values, types and locations of the neuron-to-neuron connections might be a way to find patterns of neuron communications and their restructuring during learning or passing behavioral tests (Table 2).

### 3.4. Random Neuronal-Activity Shuffling

**Figure 7:**
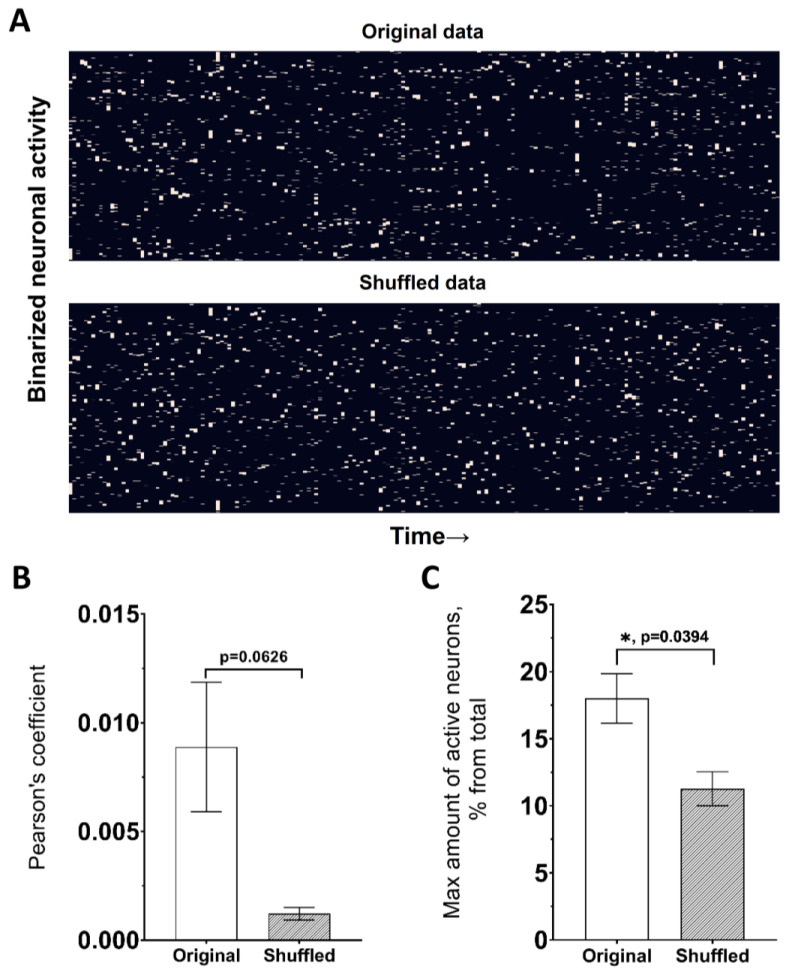
Shuffling module for variance estimation in the neuronal-activity data. (**A**) Presentation of the neuronal network in binarized form for original data (top) and shuffled with 1.0 ratio (bottom). (**B**) Pearson’s coefficient value for original and shuffled data. (**C**) Maximal amount of active neurons in 1 s (Network spike peak) for original and shuffled data. Data are presented as mean ± SEM; *: *p* < 0.05, Student’s *t*-test.

This module is designed to analyze the regularity of the statistical metrics and to verify that the obtained results are not simply due to the random signal distribution in the network, but rather reflect network characteristics. The number of activations for each neuron is kept constant, while the duration of active states and intervals are determined randomly. Additionally, there is the option to select the degree of mixing—shuffle ratio. Increasing the value of shuffling episodes utilizing the parameter “*num_of_shuffles*” in the notebook (Supplementary Materials Section S5) provides greater confidence in the results. The module displays statistical values such as the mean correlation range for a preset number of iterations, which represents the difference between the highest and lowest values of Pearson’s coefficient for each recording and the mean correlation value. The “Truth” and “False” indicators under the shuffled data show whether the placement of cells was taken into account. Metrics that describe neuronal activation parameters such as the *network spike rate* and *network spike peak* were also presented for shuffling options. For example, Figure 7A demonstrates a neuronal network map in its original state and after shuffling with a shuffle ratio of 1. The mean correlations and mean network spike peak were also represented in the same figure (Figure 7B,C). There was a significant difference between the original value of the *network spike peak* metric and the shuffled one (*p* = 0.0394, *n* = 3, Student’s *t*-test), and a visible decreasing trend in the mean value of Pearson’s correlation coefficient (*spike* method) in the mixed data, although it did not reach an appropriate level of significance (*p* = 0.0626, *n* = 3, Student’s *t*-test).

Comparing the values of statistical metrics obtained from the original and mixed data, a decrease in the average value of each presented statistical metric was observed. This is likely because shuffling the data results in a loss of information about the connections between neurons, which determines the initial values of the comparable metrics (Table 2). Thus, it can be assumed that the recorded changes in the intracellular calcium concentration, which correlated with neuronal excitation and presented statistical metrics, are biologically significant, determining the functioning of the neural network.

### 3.5. Principal Component Analysis of Statistical Metrics

**Figure 8:**
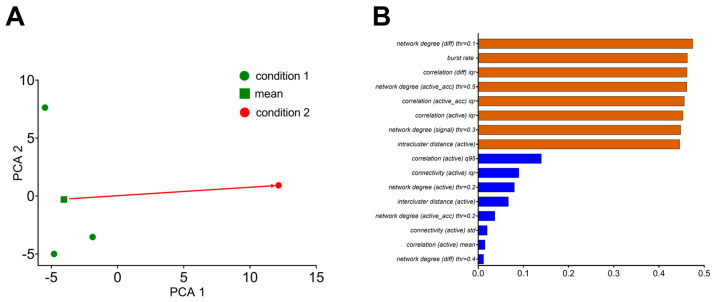
PCA dimensionality reduction method applied to obtained statistics. (**A**) Visualization of the results after applying the principal component method to reduce the dimension of the computed statistics for 3 recordings under the same experimental conditions (in green) and one recording in the state X. (**B**) Statistical metrics that make the greatest and least contributions to the PCA method.

The “Dimensionality reduction” module was developed to display a large number of the statistical metrics presented above in a two-dimensional form. It uses the PCA (principal component analysis) method to reduce the dimensions of the data and create a two-dimensional representation (Supplementary Materials Section S6). This can be helpful for the subsequent clustering of neuronal network activity, for example, by being in the different states (normal conditions/pathological, etc.) (Table 1 and Table 2). The “Dimensionality reduction” module consists of several sections: PCA results are presented as a set of values for each recording (Figure 8A), and coordinates for each recording are presented in the step below, with values for “X” and “Y” axes. Then, the top eight most and least significant features are presented as a graph (Figure 8B) and numerically. All the statistical metrics used in the study were included in the PCA analysis, and their possible biological interpretations can be found in Table 2.

The user can also specify the “state” in which the most and the least important will be displayed. The module saves all the feature values in the “.xlsx” file. In addition, the most significant features are presented using cosine similarity. For primary statistical analysis, the “Statistics and Shuffling” module was added (Supplementary Materials Section S7).

## 4. Discussion

The use of miniature fluorescence microscopy for the investigation of neuronal activity of the brain areas in freely moving animals in vivo has provided valuable comprehension in neuronal network functioning [29,30,31,32]. Analysis of calcium-indicator fluorescence helps in the evaluation of a single-neuron activity within large neuronal populations. This manuscript presents NeuroActivityToolkit, an open-source toolbox that enables the computation of various quantitative parameters and metrics describing neuronal networks (Table 1 and Table 2), based on the miniscope data preprocessed using Minian [19] or other algorithms with a similar file organization [20,21,33].

Understanding the properties of neuronal activations can be a powerful tool for analyzing the state of networks. It can find implementation in comparing neuronal network conditions under various exposures or stimuli [34,35,36], identifying shifts in the neuronal firing at the population level during learning processes [37,38,39], social behavior [40,41], seizures [42,43,44,45], etc. Analyzing the burst-rate distribution could report the excitation–inhibition balance, which is very important for proper brain functioning. The excitation–inhibition disbalance has been observed in different neurodegenerative disorders [46,47], making its investigation utilizing the miniscope technique with subsequent data analysis via NeuroActivityToolkit a promising avenue for research. Furthermore, recording neuronal calcium dynamics with specific promoters for selective expression in the neuronal subtypes (CAMKII for most excitatory neurons [48,49] and mGAD65 [50] or others [51] for inhibitory neurons) might be a way to reveal the neuronal structure architecture of the complex networks in vivo in different regions of the brain.

Correlation coefficient analysis offers a comprehensive understanding of the connectivity between cells in the neuronal network [52,53]. Tracking the dynamics of the co-active neuronal pairs might be an interesting approach for plasticity-related processes investigation. The appearance of new pairs of strongly correlated neurons and the disappearance of already-formed ones, as well as the redistribution of Pearson’s correlation coefficient among a neuronal network, may indicate a complex reorganization within the neural network in response to external factors. Pearson’s coefficient calculation for different variants of the active-phase extraction from the preprocessed calcium signal is implemented in the NeuroActivityToolkit, providing a complete picture of the “relationships” between neurons in the network. Considering the spatial position of the correlated neuronal pairs allows users to assess the redistribution of connections between neurons in space. Moreover, the emergence of new interactions can shed light on how the network rebuilds in response to external influences or pathological changes, such as strokes, epileptic seizures, or during the progression of neurodegenerative diseases.

In the current toolbox, the shuffling module helps to determine the level of (un)randomness in the recorded datasets. Data shuffling is an important tool in studying the neurobiological aspects of the brain’s neural networks’ functioning [54,55]. By introducing controlled randomness to the original data, neuronal connections between cells are eliminated along with changes in the overall activity of the neural network. Evaluation of the statistical metrics obtained from both original data and shuffled data offers the establishment of the levels of certainty in the neural circuits. This undoubtedly plays a significant role in fundamental aspects of neuronal network functioning.

Principal component analysis can be useful for clustering neural networks by their calculated statistical parameters, with a convenient graphical presentation for various conditions and to track their changes.

The presented toolbox NeuroActivityToolkit is a valuable tool for the high-level analysis of miniscope data, which allows neurobiologists to determine and compare quantitative metrics for describing neuronal networks.

## Figures and Tables

**Table 1:** Parameters and metrics that were implemented for the quantitative miniscope data analysis in the NeuroActivityToolkit toolbox.

No.	Parameter	Meaning	Module Location
1	spike	Method for the active-phase detection as a sharply rising stage of calcium-indicator intensity	ActiveStateAnalyzer
2	full	Method for the active-phase detection as a part above calculated threshold value	ActiveStateAnalyzer
3	signal	Method for the detection of the active phase as a whole initial signal of intensity (applicable to Pearson’s coefficient calculations and other connected metrics and distance analysis)	ActiveStateAnalyzer, Distance analysis
4	warm	Minimal duration of the fluorescence-signal passive phase (can be varied from 0 to 100), number of frames	ActiveStateAnalyzer
5	cold	Minimal duration of the fluorescence-signal active phase (can be varied from 0 to 100), number of frames	ActiveStateAnalyzer
6	window	Configurable value for fluorescent signal smoothing, number of pixels	ActiveStateAnalyzer
7	burst rate	Number of “cell activations” for a set period of time, percent of neurons with the given number of activations	ActiveStateAnalyzer
8	single neuron “activations”	Number of single neuron activations per minute (can be obtained via saving the *Burst rate* as a “.xlsx”), number of activations per minute	ActiveStateAnalyzer
9	network spike rate	Percent of active neurons over a certain period of time, %	ActiveStateAnalyzer
10	network spike peak	Maximal number of simultaneously active cells for a certain period of time, %	ActiveStateAnalyzer
11	network spike duration	Time length in which the number of simultaneously active cells is higher than the preset threshold value, percent of total time	ActiveStateAnalyzer
12	Pearson’s coefficient of correlation	Calculated for the intensity of the original signal(*signal)*, the intensity derivative (*diff)*, binary results of active-phase segmentation (*active* or *full)* method, coefficient of linear pairwise correlation, and connection of intersection (*active_acc*, *relation of the simultaneously active states duration to the sum of the both of neurons activity time)*	ActiveStateAnalyzer, Distance analysis
13	lag	Maximal delay value between neuronal activations, frames	ActiveStateAnalyzer
14	network degree	Percent of co-active neuronal pairs above the threshold level, %	ActiveStateAnalyzer
15	connectivity	Distribution of the connectivity shares for each neuron, %	ActiveStateAnalyzer
16	mean correlation range	Difference between the maximal and minimal value of correlation	MultipleShuffling
17	rho	Distance to neurons from the center of their mass in polar coordinates, pixels	Distance analysis
18	Euclidean	Distance between co-active neuronal pairs, pixels	Distance analysis
19	radial	Difference in distances between co-active pairs of neurons from the mass center of all neurons for recording, pixels	Distance analysis
20	transfer entropy	The entropy of transfer from neuron X to another neuron Y is the amount of uncertainty reduced in future values of Y by knowing the past values of X, providing the corresponding past values of Y (metric to apply or not for PCA analysis (step 2))	Dimensionality reduction

**Table 2:** Statistical metrics implemented in the NeuroActivityToolkit toolbox and their proposed biological interpretation.

No.	Statistical Metric	Possible Biological Interpretation
1	single neuron “activations” and burst rate	Describes a total number of neuronal activations at the single-cell level and as a total activity of the whole network. It can be used for the comparison of the neuronal network state, in particular conditions or pathological states, for validation of the hypo- or hyperactivation profile of the brain region. Also, it can be used as a trivial marker of agonist/antagonist action on neuronal excitation levels.
2	network spike rate	Neuronal network excitation levels could be described by these metrics. Analyzing shifts in the distributions might provide complex information about changes in the firing rate of all neurons that are part of the network. It is a more sophisticated and informative way to validate differences in activation profiles observed in the distinct area of the brain, which is often affected by various pathologies.
3	network spike peak	
4	network spike duration	Time duration in which more than a set percent of neurons was active in the neuronal network. This metric is tightly bound to the ones mentioned above; nevertheless, it explicitly reflects an elongation/reduction in the total neuronal activity duration, which might indicate changes in the excitation or elevation/decrease in the synchronically firing pattern shifts of the distinct brain region.
5	Pearson’s coefficient of correlation	The similarity in the activation patterns between neurons can be reflected as a correlation coefficient. On the one hand, the disruption of the synaptic plasticity processes is a hallmark of various neuropathologies, for example, neurodegenerative diseases. Correlation coefficient evaluation with changing levels of strictness might be a promising way to determine early changes in the prodromal stage of diseases. On the other hand, processes of learning, adaptation, etc., are also connected with pairwise neuronal correlations as new pairs appear and others vanish. Such reorganization might be possibly expressed in the elevation or decrease in the mean value of Pearson’s coefficient with a set threshold value.
6	network degree	
7	shuffled neuronal activity	This module is performed to determine the regularity of the statistics obtained (they have a biological/physiological nature) or if they are random variables. In this module, the number of activations is kept constant for each neuron, while the duration of active states and the duration between them are determined randomly.
8	distance between coactive neurons (Euclidian or radial)	The evaluation of the reorganization of the neuronal network during applied stimuli or specific conditions. Investigation of the architecture of neuronal coactive pairs and its regularity for defined areas of the brain.
9	principal component analysis applied to calculated metrics	PCA method for obtained statistical-metric clustering for determining differences in the total neuronal network state as a response to external shifts, processes of learning, etc. Might be a powerful tool for early-stage estimations of changes during pathological processes at the total neuronal network level.

## Data Availability

The “NeuroActivityToolkit” toolbox is deposited in an online repository, accessible by the following link: https://github.com/spbstu-applied-math/NeuroActivityToolkit (accessed on 19 September 2023) and https://appliedmath.gitlab.yandexcloud.net/lmn/miniscope (accessed on 19 September 2023). The example dataset contains three raw recordings in “.avi” format at a rate of 20 frames per second for the same 9-month-old FVB-background male mouse.

## Supplementary Materials

The following supporting information can be downloaded at: https://www.mdpi.com/article/10.3390/jimaging9110243/s1, Figure S1: Parameters influencing the active-phase determination; Figure S2: Methods for calculation of the correlation coefficient implemented in NeuroActivityToolkit, with a preset threshold level at 0.3. Video S1: Activity of the hippocampal neuronal network recorded via miniscope (recording 1); Video S2: Activity of the hippocampal neuronal network recorded via miniscope (recording 2); Video S3: Activity of the hippocampal neuronal network recorded via miniscope (recording 3). Detailed tutorial can be found using a link above.
