# Supplementary material for: NeuroActivityToolkit—Toolbox for Quantitative Analysis of Miniature Fluorescent Microscopy Data
Source: J Imaging. 2023 Nov 6;9(11):243. doi: 10.3390/jimaging9110243 (PMC10672520; doi:10.3390/jimaging9110243)
Supplement: Supplementary file 1 [file jimaging-09-00243-s001.zip › Supplementary Materials (figures).pdf]

Supplementary Figures

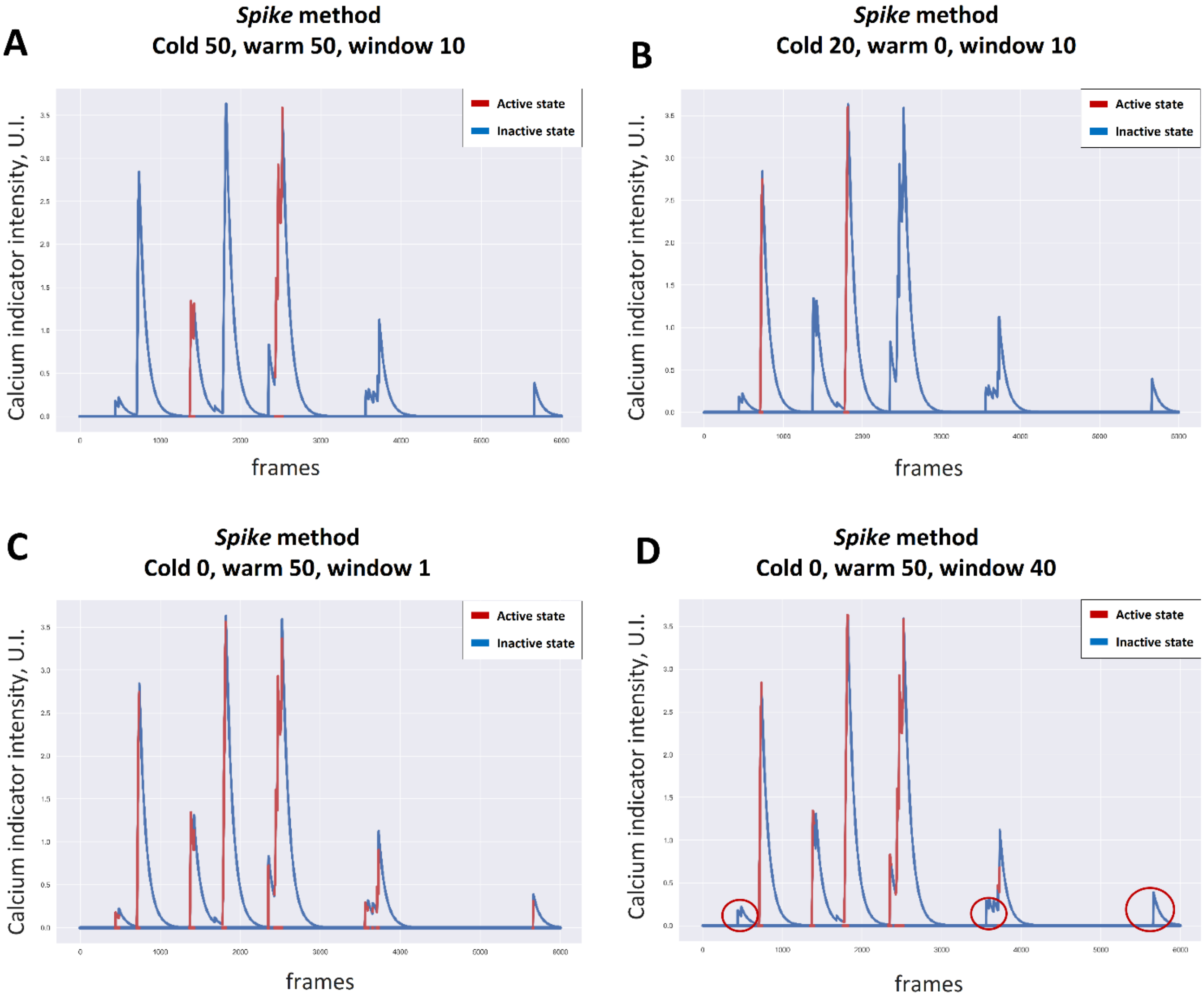

Supplementary Figure S1. Parameters influence on the active phase determination. **(A, B)** Influence of the *warm* and *cold* parameters for segmentation of the rising phase of the calcium indicator fluorescence. **(C, D)** *Window* parameter value is important for determining active phase for small-magnitude fluorescent changes of the calcium indicator. By red circles in the **(C)** disappeared active states in comparison to **(D)** value are mentioned.

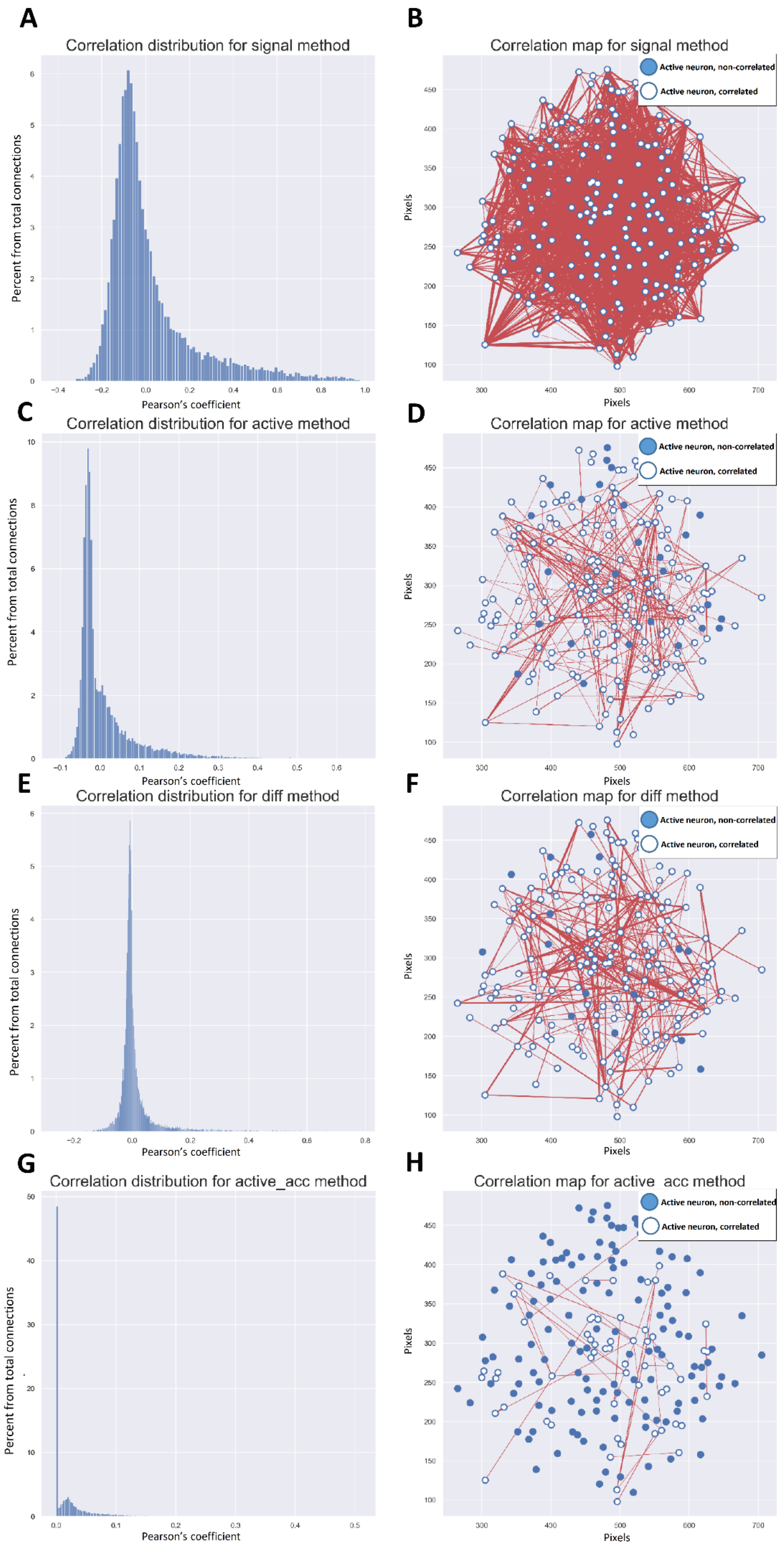

A
Correlation distribution for signal method
Percent from total connections
Pearson's coefficient
B
Correlation map for signal method
Active neuron, non-correlated
Active neuron, correlated
Pixels
Pixels
C
Correlation distribution for active method
Percent from total connections
Pearson's coefficient
D
Correlation map for active method
Active neuron, non-correlated
Active neuron, correlated
Pixels
Pixels
E
Correlation distribution for diff method
Percent from total connections
Pearson's coefficient
F
Correlation map for diff method
Active neuron, non-correlated
Active neuron, correlated
Pixels
Pixels
G
Correlation distribution for active_acc method
Percent from total connections
Pearson's coefficient
H
Correlation map for active_acc method
Active neuron, non-correlated
Active neuron, correlated
Pixels
Pixels

Supplementary Figure S2. Methods for calculation correlation coefficient implemented in NeuroActivityToolkit with preset threshold level at 0,3. **(A)** Correlation coefficient distribution for *signal* method. **(B)** Correlation map for *signal* method. **(C)** Correlation coefficient distribution for *active* method. **(D)** Correlation map for *active* method. **(E)** Correlation coefficient distribution for *diff* method. **(F)** Correlation map for *diff* method. **(G)** Activity intersection distribution for *active_acc* method. **(H)** Correlation map for *active_acc* method.
