# Supplementary material for: NeuroActivityToolkit—Toolbox for Quantitative Analysis of Miniature Fluorescent Microscopy Data
Source: J Imaging. 2023 Nov 6;9(11):243. doi: 10.3390/jimaging9110243 (PMC10672520; doi:10.3390/jimaging9110243)
Supplement: Supplementary file 1 [file jimaging-09-00243-s001.zip › Supplementary Materials (tutorial).pdf]

Supplementary Material

Tutorial

1. Installation

System requirements:

- Windows 8 or newer
- Minimum 1GB RAM
- Minimum 6 GB disk space

1.1. Installation steps:

Download and install the minian package from: https://github.com/denisecailab/minian;
Download and unzip the code folder from: https://github.com/spbstu-applied-math/NeuroActivityToolkit or https://appliedmath.gitlab.yandexcloud.net/lmn/miniscope.
Install Anaconda and open Anaconda Promt;
Execute:

```
cd PATH_TO_CODE
conda create --name neuron-analysis -c conda-forge --file requirements.txt –y
```

1. To run the software open Anaconda Promt, execute:

```
cd PATH_TO_CODE
conda activate neuron-analysis
jupyter notebook
```

After this steps, the joined Jupiter Notebook, containing separated notebooks, will be opened:

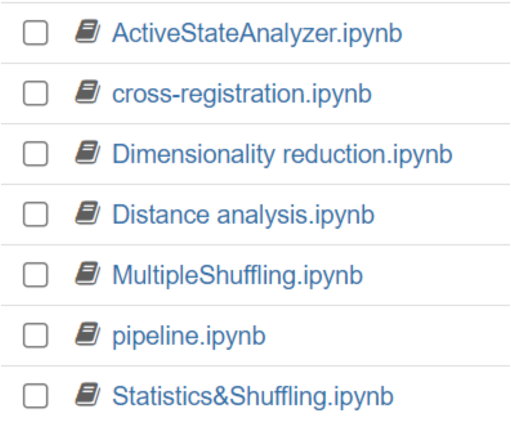

Click on required notebook to start. Notebooks descriptions are provided below.

2. Example dataset

Example dataset contains of 3 recording for the same FVB mouse aged 9 months. The data is raw recordings for different days in ".avi" format that are ready to be processed via "Minian".

3. "ActiveStateAnalyzer" module

This Notebook is used for processing the results of the "Minian" software package or others with the same output. The main role of this module is to compute active states of the individual neurons by different methods and to obtain metrics that are connected to activation parameters of the neurons.

Parameters:

Params

Ввод [3]:
```
# Path to minian output
path_to_data = 'demo_movies/mouseold/2/minian/'
```

Ввод [4]:
```
verbose = True
```

Ввод [5]:
```
fps = 20
```

In this step parameters of miniscope data are set:

- path_to_data – a path to the processed data via “Minian”;
- verbose – define visualization of progress bars;
- fps – frames per seconds for recording (important to set right value as all metrics are based on this value).

3.1. Active state of neuron determination

Setting parameters for detecting the active state of a neuron:

- neuron – drop-down list with neuron selection;
- method – a way to detect active phase:
  - spike – only a stage of rapid intensity growth,
  - full – a stage of growth and decline of the intensity which is higher than calculated threshold value.
- cold – minimum duration of the active phase;
- warm – minimum duration of the passive phase;
- window – width of the moving average smoothing window;
- Set parameters –setting the selected parameters and applying them to all neurons (highly important to “set parameters”, otherwise further steps won’t work).

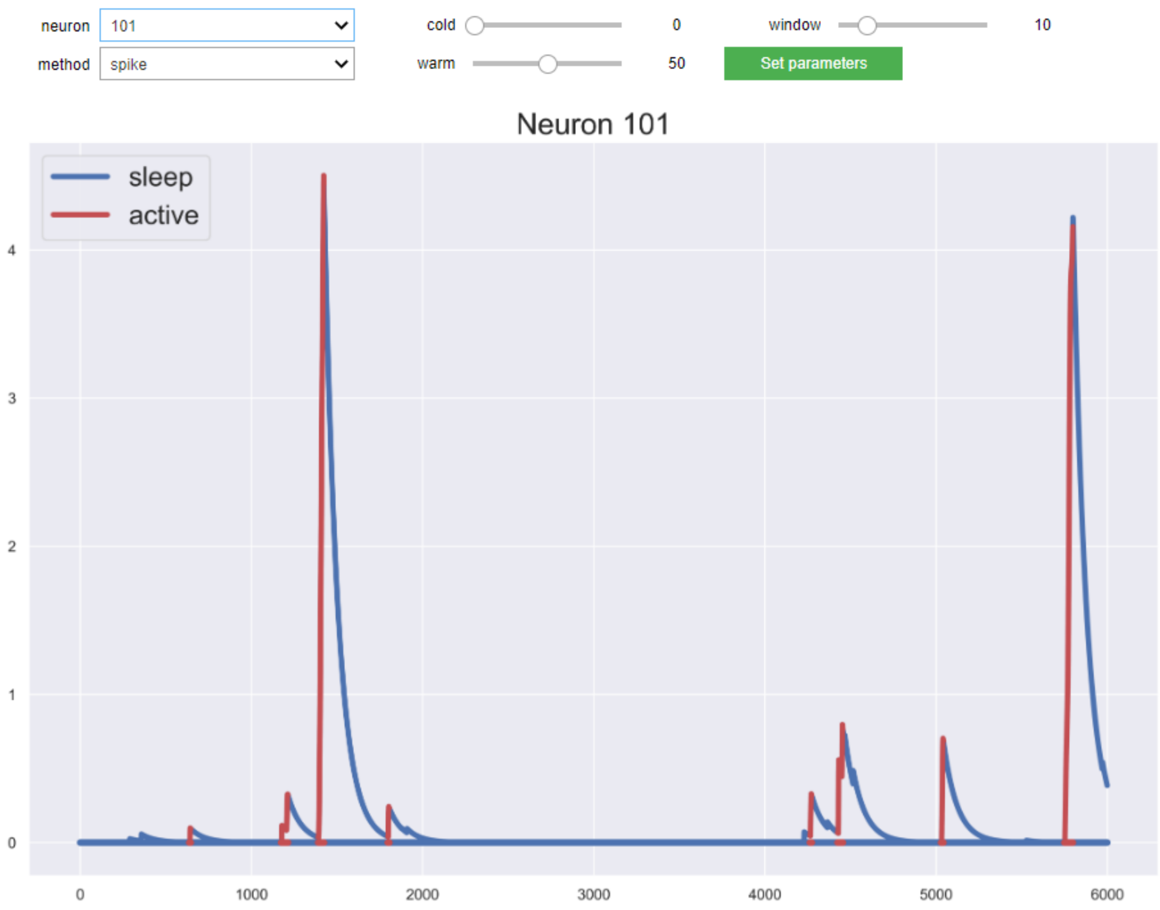

3.2. Burst rate

Burst rate – the number of "cell activations" for a given period of time.

The distribution of burst rate values is visualized.

- max_bins – maximum number of columns on the chart;
- Save – saving the distribution in “.xlsx” format.

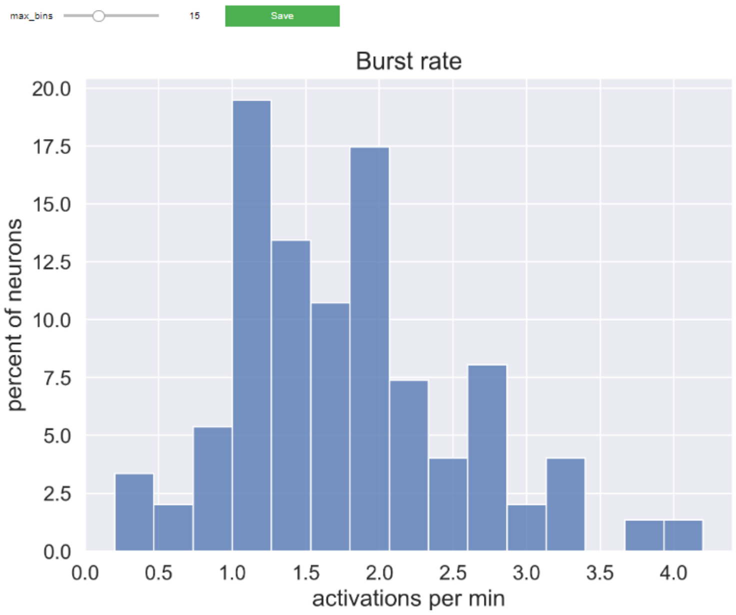

3.3. Network spike rate and Network spike peak

Network spike rate – the active neurons percent in the interval of time;

Network spike peak – the maximal number of active cells for all the period of the recording with a certain inter-val of time.

- period – duration of the interval for which metric will compute results (1sec-60sec);
- Save – saving the distribution in ".xlsx" format.

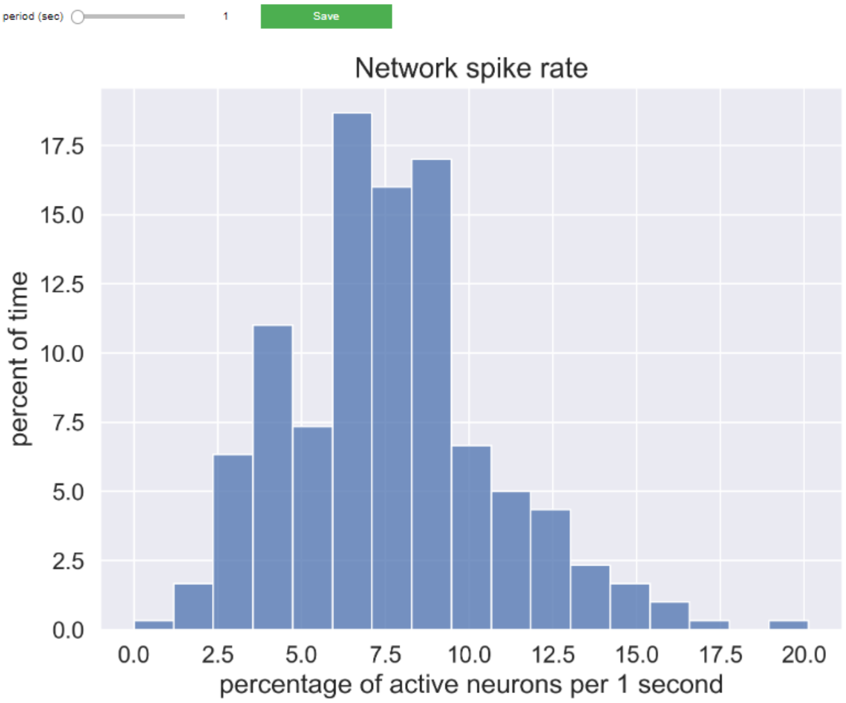

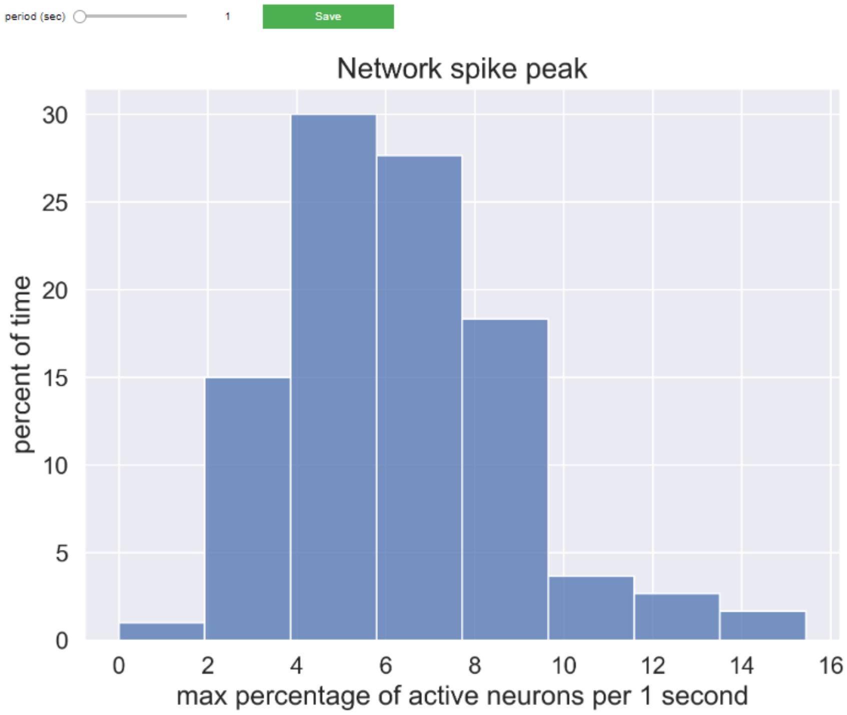

3.4. Network spike duration

Network spike duration is a time duration when the number of active cells is higher than the predetermined threshold value.

- Thresholds – a variable for individual preset threshold value of simultaneously active neurons.
- Save – saving the distribution in ".xlsx" format.

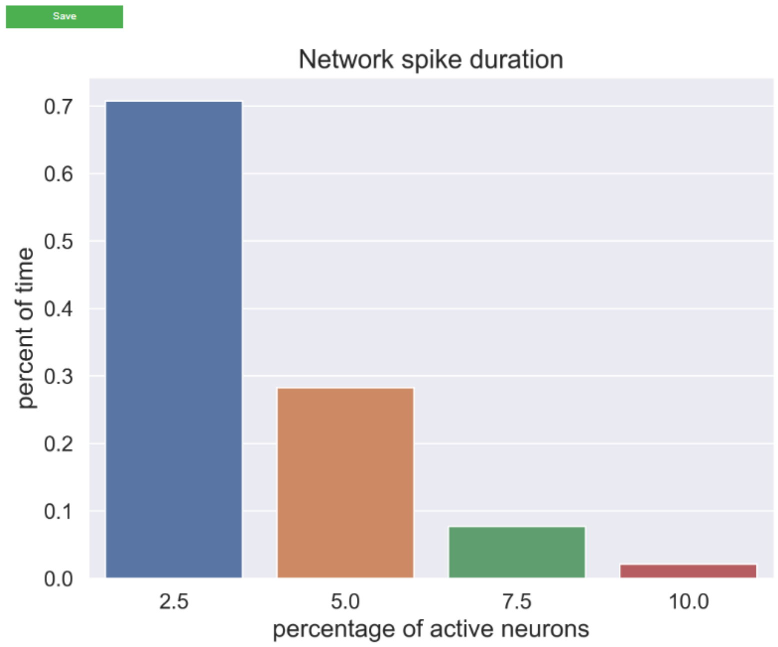

3.5. Correlation

- Choosing one of the possible types of interconnection between neurons:
  - signal – Pearson's correlation coefficient for the intensity of the original signal,
  - diff – Pearson's correlation coefficient by intensity derivative,
  - active – Pearson's correlation coefficient based on binary results of active phase segmentation,
  - active_acc – the connection of intersection to the union of active states of neurons,
  - transfer_entropy – entropy of transfer between neurons.
- position – taking into account the spatial distance between neurons;
- threshold – threshold value for visualization;
- lag – maximal delay value between neuronal activations.

  maximum time shift of neurons relative to each other.

Automatically saved into "results".

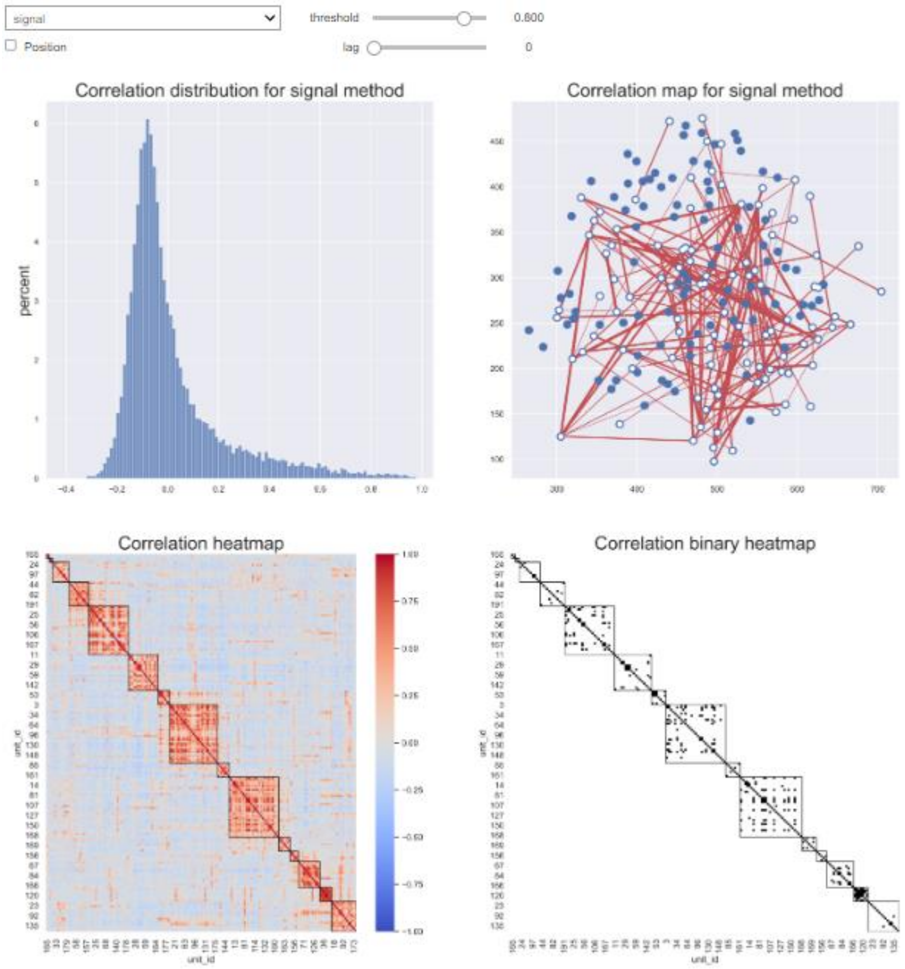

3.6. Network degree

Network degree –percent of co-active neurons above the threshold level;

- Choosing one of the possible types of interconnection between neurons:
  - signal – Pearson's correlation coefficient for the intensity of the original signal,
  - diff – Pearson's correlation coefficient by intensity derivative,
  - active – Pearson's correlation coefficient based on binary results of active phase segmentation,
  - active_acc – the connection of intersection to the union of active states of neurons,
  - transfer_entropy – entropy of transfer between neurons.
- Save – saving results as ".xlsx" format.

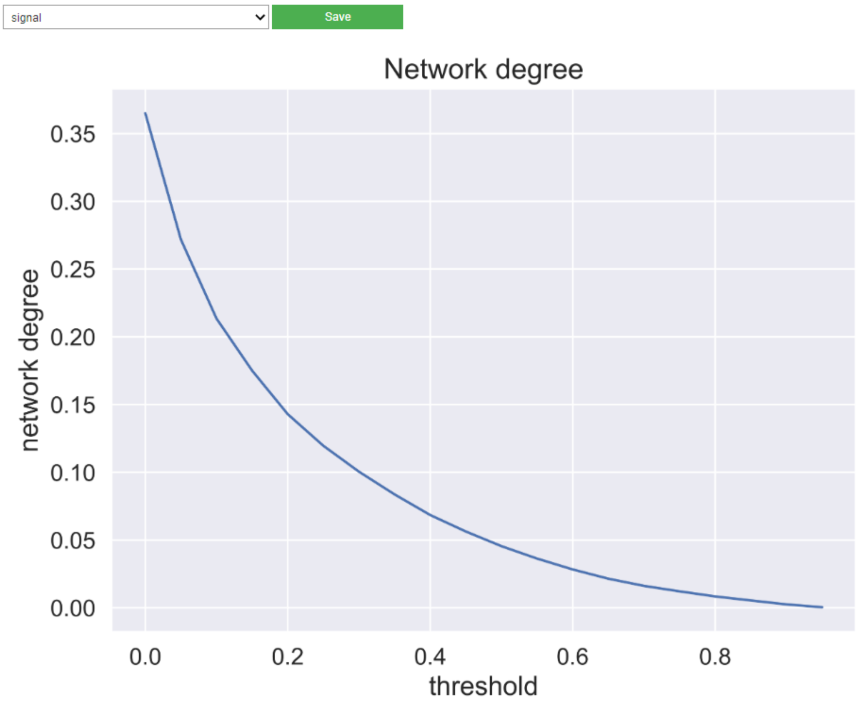

3.7. Connectivity

Connectivity – distribution of the connectivity share for each neuron.

- Choosing one of the possible types of interconnection between neurons:
  - signal - Pearson correlation coefficient for the intensity of the original signal,
  - diff – Pearson correlation coefficient by intensity derivative,
  - active – Pearson correlation coefficient based on binary results of active phase segmentation,
  - active_acc – the connection of intersection to the union of active states of neurons,
  - transfer_entropy – entropy of transfer between neurons.
- threshold – minimal correlation value.
- Save – saving results as ".xlsx" format.

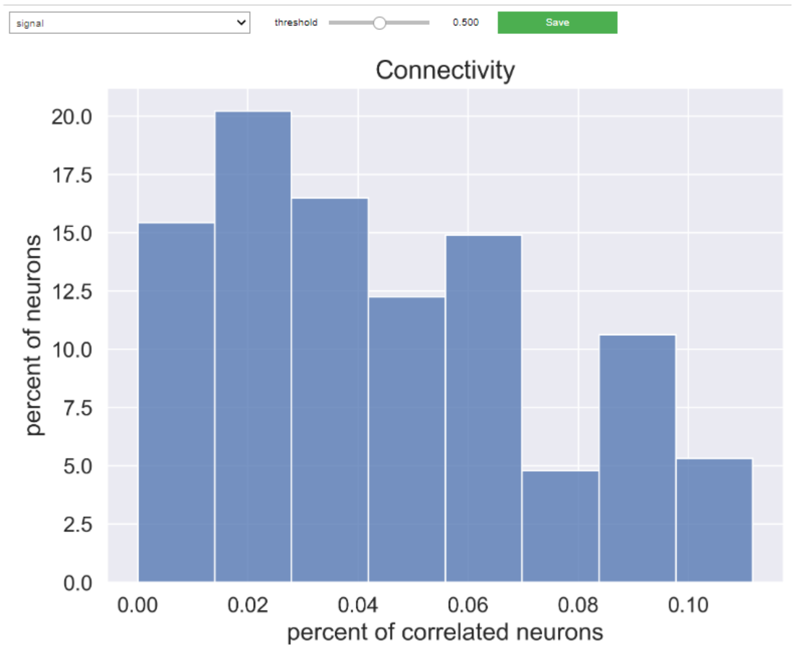

“ActiveStateAnalyzer” notebook **must be run first of all** because all the calculated statistics are used in the futher modules. All the results for each recording are saved into folder“results” as stated in the “path_to_data”.

4. Distance analysis

“Distance analysis” notebook is created for evaluating the distance criteria for pairwise correlations between neurons.

- path_to_data – the path to the root directory with the data;
- dates – information about all the recordings of interest:

```
dates = [
    '1',
    '2',
    '3'
]

path_to_data = 'demo_movies/mouseold'

fps = 20
```

If the path stated correctly, graphs will be shown with possibility to save them in “.xlsx”. When calculating the average distances between pairs of coactive neurons, user has the option to choose between the "Euclidean" and "radial" representation.

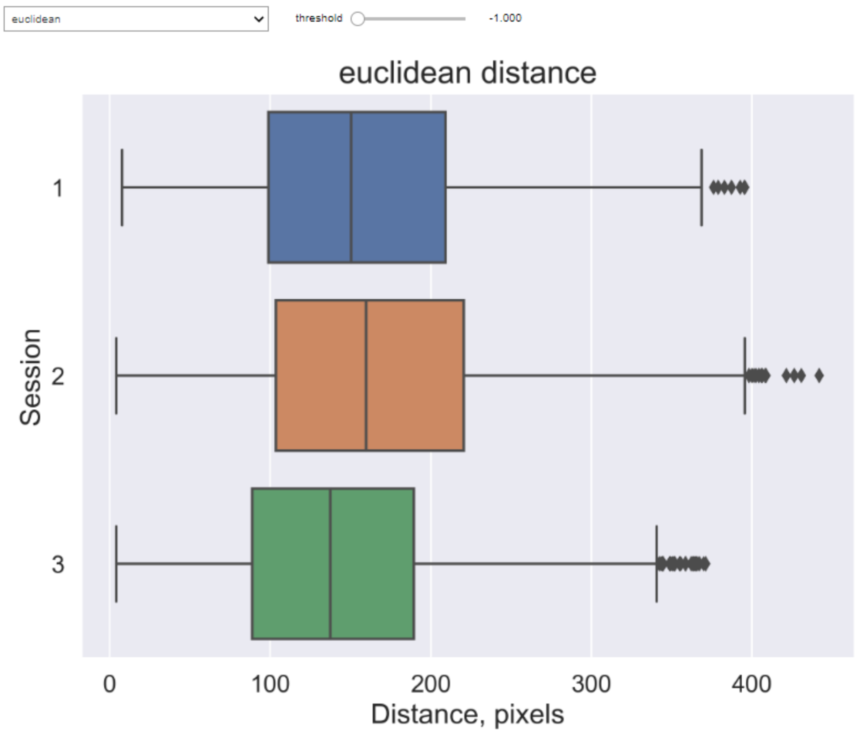

Metrics of interest can be set for visualization by widgets.

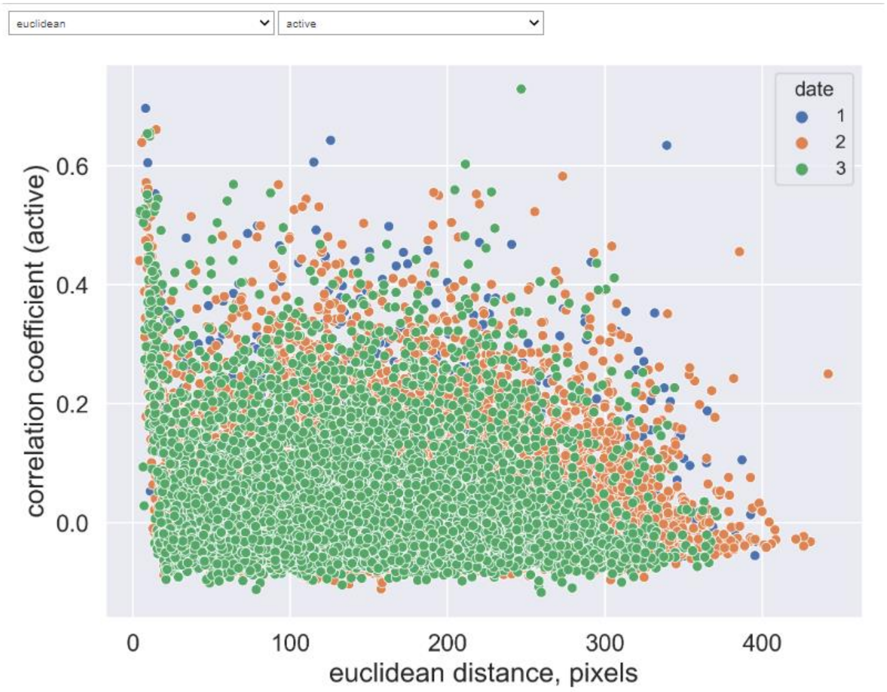

All the presented metrics can be saved in the file with ".xlsx" format for further analysis, comparison and visualization.

5. Shuffling module ("MultipleShuffling")

This notebook is created for shuffling the miniscope data to define level of variance and for comparison statistics from "original" data with "shuffled" one.

- path_to_data – the path to the root directory with the data;
- dates – information about all the recordings of interest:

- key – title of the recording, should be unique;
- values:
  - path – the path to the data relative to the root directory,
  - mouse – mouse identificator,
  - condition – condition of the mouse while recording,
  - fps – frames per second,

- num_of_shuffles – amount of the shuffling events. Usually for sufficient amount of recordings value of “1” is used. Can be increased for more robustness;
- shuffle_fractions – shuffle ratio for mixing module (can be manually changed).

```
dates = {

  '1': {'path': 'mouseold/1',
                'mouse': '1',
                'condition': 'normal',
                'fps': 20},
    '2': {'path': 'mouseold/2',
                'mouse': '1',
                'condition': 'normal',
                'fps': 20},
    '3': {'path': 'mouseold/3',
                'mouse': '1',
                'condition': 'normal',
                'fps': 20}
  }
path_to_data = 'demo_movies'

num_of_shuffles = 4

shuffle_fractions = [.5, 1.]
```

Shuffled data are saved into path_to_data folder as individual “.xlsx” tables.
Here, mean correlation range (difference between the biggest and lowest value of Pearson’s coefficient for each recording for active method) calculated for original data (Shuffle ratio equals 0), and shuffled data with shuffle ratio 0,5 and 1.

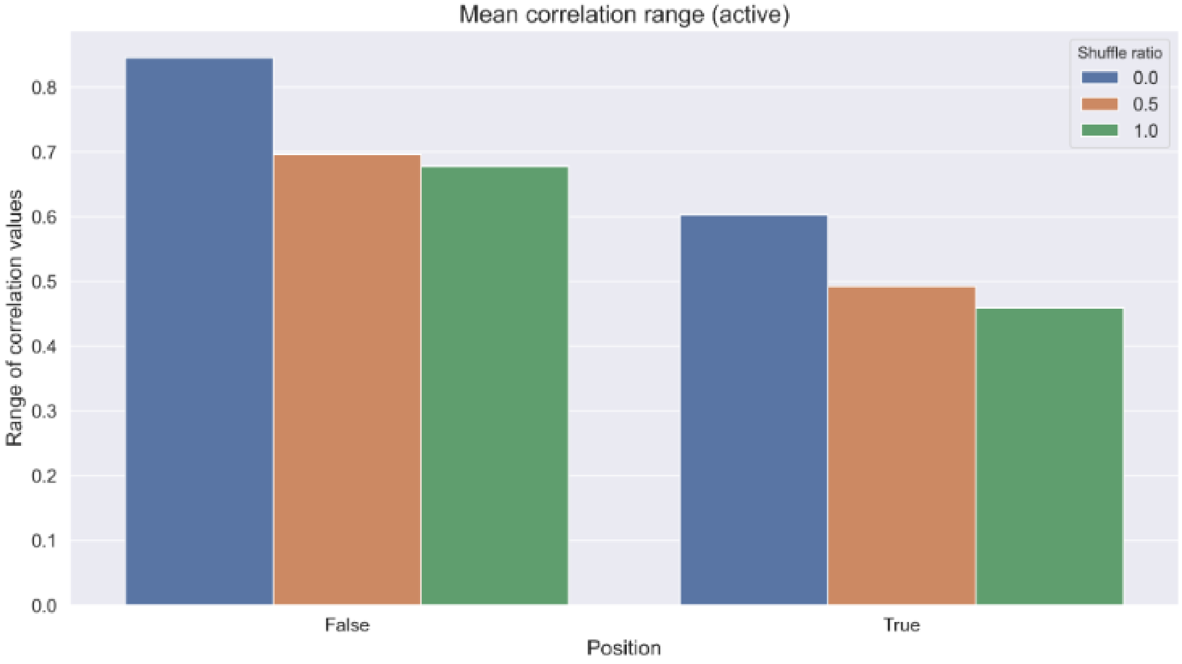

Calculation of the Network spike peak statistical metric on the original and shuffled data.

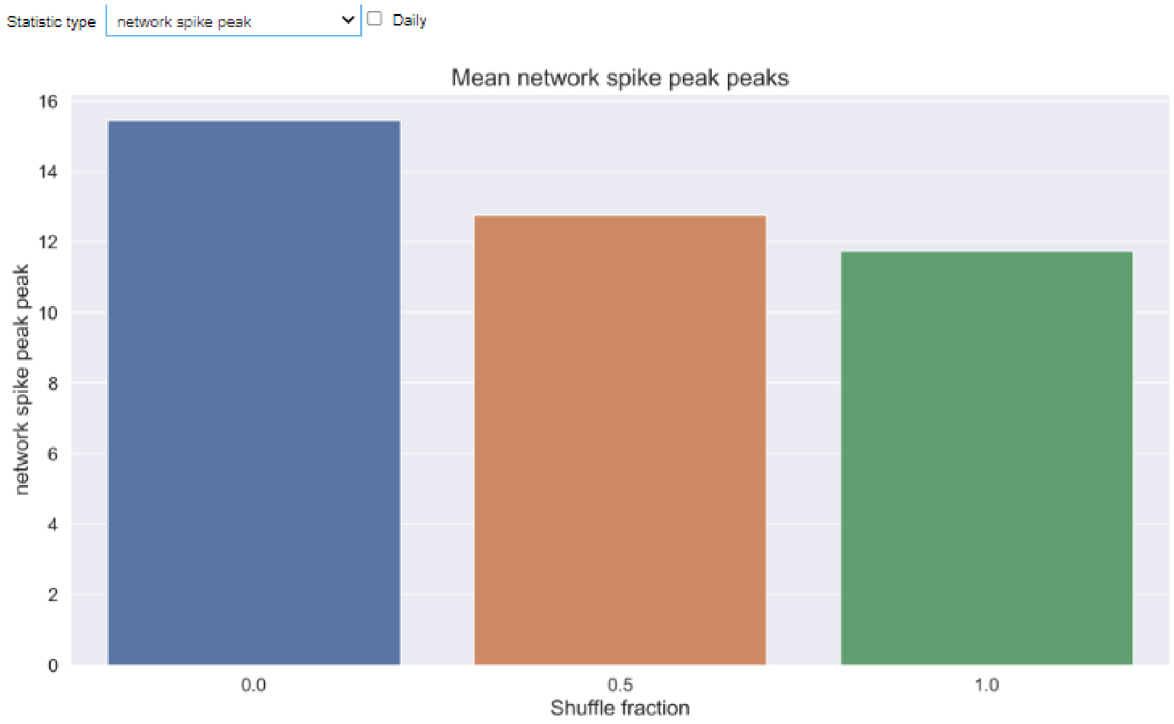

6. “Dimensionality reduction” module

6.1. Data

- path_to_data – the path to the root directory with the data
- transfer_entropy – using transfer entropy or not (when using much more time is needed for calculations);
- dates – information about all the recordings of interest:
    - key – unique record identificator (It must be unique for each mouse, as in example ‘mouseold1’, ‘mouseold2’ etc.),
    - values:
        - path – the path to the data relative to the root directory,
        - mouse – mouse identificator,
        - condition – condition of the mouse while recording,
        - fps – frames per second;
- conditions_order – chronological order for recordings;
    - key – unique identificator of the mouse (the same as in “dates”),
    - values – chronological order of recordings.

```
path_to_data = 'demo_movies'

transfer_entropy = False

dates = {

'mouseold1': {'path': 'mouseold/1',
                  'mouse': '1',
                  'condition': '1',
                  'fps': 20},
    'mouseold2': {'path': 'mouseold/2',
                  'mouse': '1',
                  'condition': '1',
                  'fps': 20},
    'mouseold3': {'path': 'mouseold/3',
                  'mouse': '1',
                  'condition': '1',
                  'fps': 20},

'mouseX': {'path': 'mouse3/right_after',
                  'mouse': '1',
                  'condition': '2',
                  'fps': 15},
}

conditions_order = {
    '1': ['1', '2'],
    }
```

6.2. PCA visualization

Visualization of results after dimensionality reduction via PCA method. The arrows visualize the chronological order specified in conditions_order.

- mouse – mouse identificator to show;

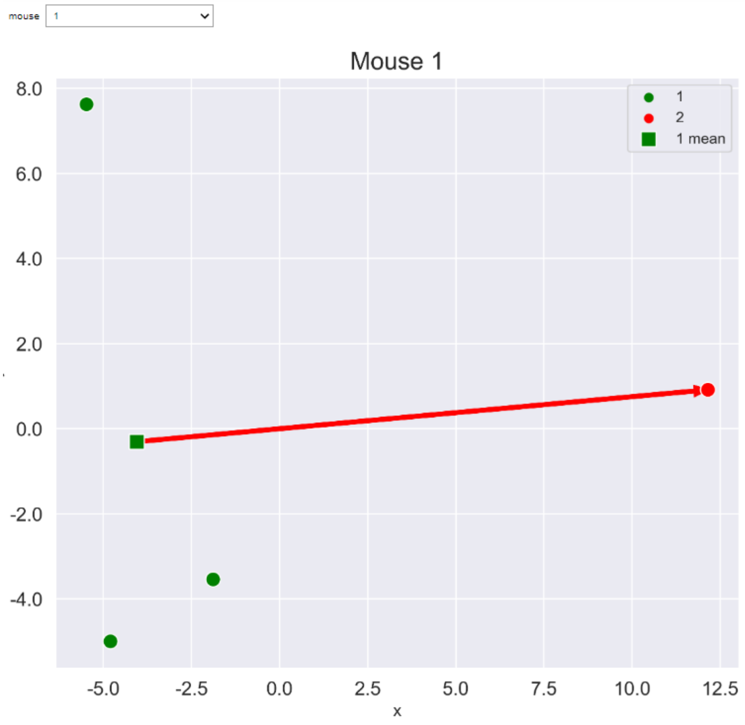

Results of the PCA method are saved into folder “path_to_data”. Also, under this window at the step “8” coordinates for each point can be found.

Visualization of statistical deviation. The top 8 most highly deviant and stable statistics are displayed.

- condition – parameter to define whether visualization is made for all the conditions or only for chosen one.

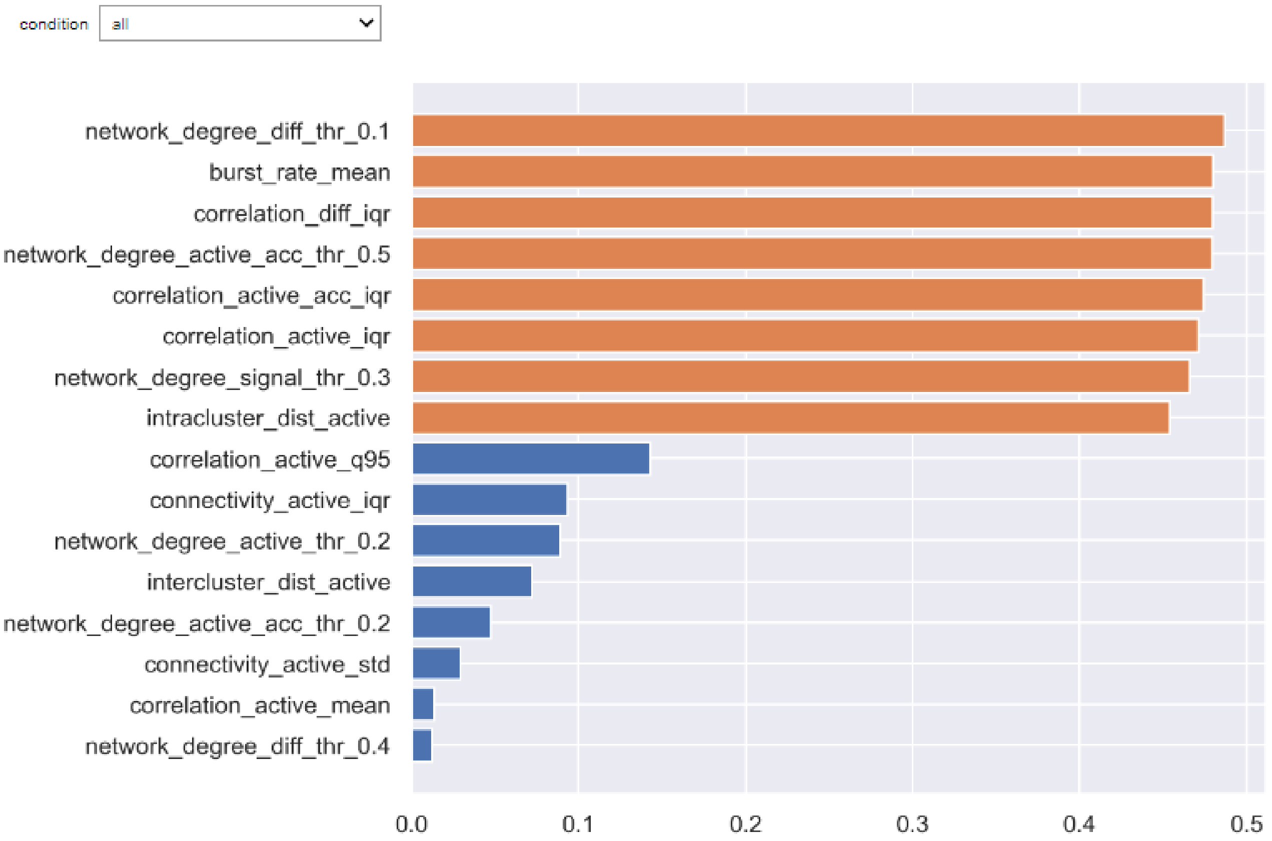

7. Statistics and shuffling

This module is done for primary statistical analysis of the obtained data. It consists of several parts that are described below.

- path_to_data – the path to the root directory with the data;
- dates – information about all the recordings of interest:
  - key – title of the recording, should be unique;
  - values:
    - path – the path to the data relative to the root directory,
    - mouse – mouse identificator,
    - condition – condition of the mouse while recording,
    - fps – frames per second,

```python
# path to the folder with the processed data
path_to_data = 'demo_movies'

# sessions params
# key - unique id
# path - path to session data
# mouse - mouse id
# condition (1/2 etc)
# fps - frames per second
dates = {
    'mouseold1': {'path': 'mouseold/1',
                  'mouse': '1',
                  'condition': '1',
                  'fps': 20},
    'mouseold2': {'path': 'mouseold/2',
                  'mouse': '1',
                  'condition': '1',
                  'fps': 20},
    'mouseold3': {'path': 'mouseold/3',
                  'mouse': '1',
                  'condition': '1',
                  'fps': 20},

    'mouseX': {'path': 'mouse3/right_after',
               'mouse': '1',
               'condition': '2',
               'fps': 15},
}
```

Further, neuronal network representation in the binarized form will be computed (for original data in the top and for shuffled data below). By the widget in the left top corner recording of the interest can be chosen.

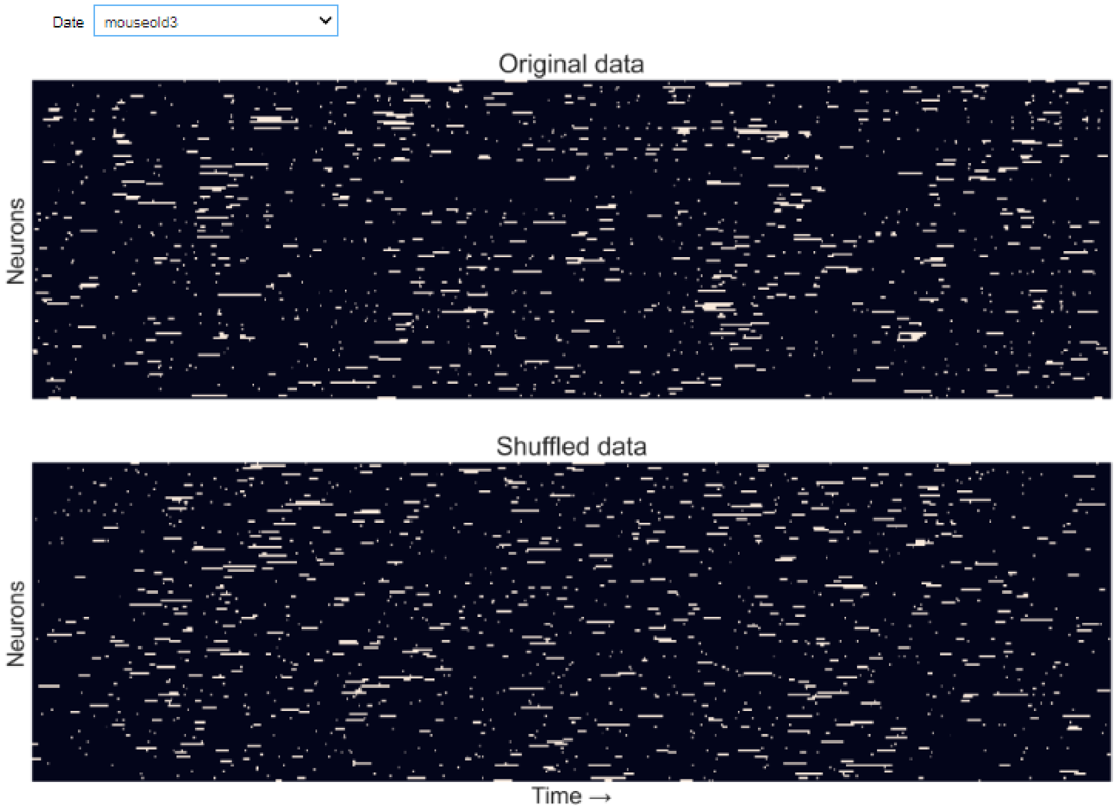

Then, statistical modules are implemented. Here variuos metrics can be compared with each other by different statistical tests. Widgets are implemted for choosing appropriate statistical test and for selection of the comparable values. Both of the right widgets are served for p-value representation ways (with simple p exact value, by stars or both together).

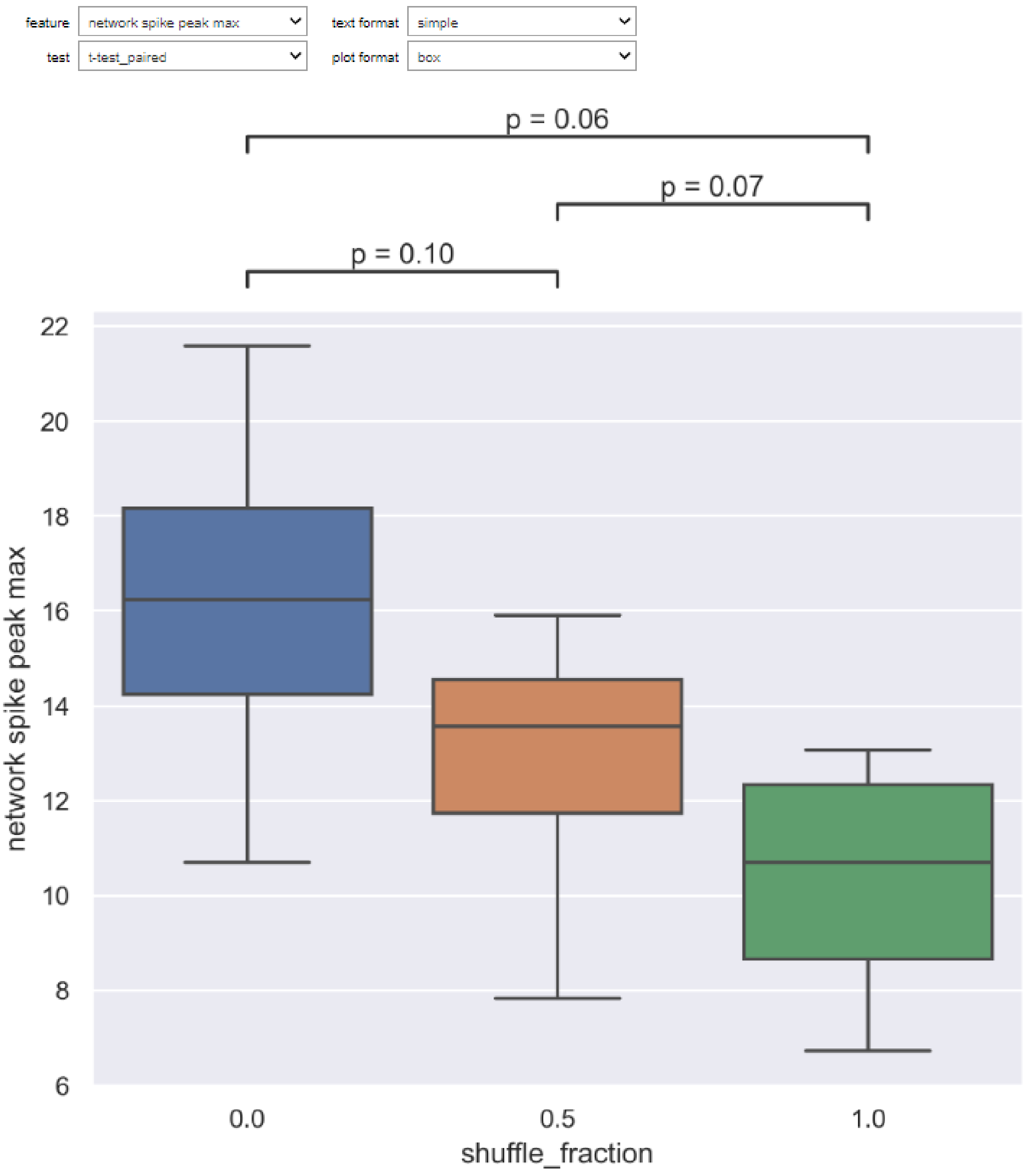

The same comparison is available for distance analysis of co-active neuronal pairs. It is possible to choose between “Euclidian” and “radial” distances.
